# Pharmacokinetics and pharmacodynamics of a long-acting monoclonal antibody against malaria in African adults

**DOI:** 10.1172/JCI207559

**Published:** 2026-07-02

**Authors:** Tuan M. Tran, Zonghui Hu, Kassoum Kayentao, Aissata Ongoiba, Sam Jones, Nada Abla, Sara A. Healy, Hamidou Cisse, Bickey H. Chang, Jeff Skinner, Leonid Serebryannyy, Sandeep R. Narpala, Robin Schlesinger, Kwang Low, Rachel Kazmierski, Bob Lin, Joana Dias, Safiatou Doumbo, Didier Doumtabe, Anne C. Preston, Shanping Li, Mary E. Peterson, Amit Oberai, Adam D. Shandling, Joseph J. Campo, Sean C. Murphy, Shinyi Telscher, Emily E. Coates, Edmund V. Capparelli, Amagana Dolo, Boubacar Traore, Robert A. Seder, Peter D. Crompton

**Affiliations:** 1 Division of Infectious Diseases, Department of Medicine, and; 2 Ryan White Center for Pediatric Infectious Disease and Global Health, Department of Pediatrics, Indiana University School of Medicine, Indianapolis, Indiana, USA.; 3 Office of Biostatistics Research, National Institute of Allergy and Infectious Diseases, NIH, Bethesda, Maryland, USA.; 4 Malaria Research and Training Centre, Mali International Center of Excellence in Research, University of Sciences, Techniques and Technologies of Bamako, Bamako, Mali.; 5 Medicines for Malaria Venture, Geneva, Switzerland.; 6 Malaria Infection Biology and Immunity Section, Division of Intramural Research, and; 7 Vaccine Research Center, National Institute of Allergy and Infectious Diseases, NIH, Bethesda, Maryland, USA.; 8 Antigen Discovery Inc., Irvine, California, USA.; 9 Department of Laboratory Medicine and Pathology and Center for Emerging and Re-emerging Infectious Diseases, University of Washington, Seattle, Washington, USA.; 10 Department of Laboratories, Seattle Children’s Hospital, Seattle, Washington, USA.; 11 Departments of Pediatrics and Pharmacy, UC San Diego School of Medicine and Skaggs School of Pharmacy and Pharmaceutical Sciences, La Jolla, California, USA.

**Keywords:** Clinical Research, Immunology, Infectious disease, Malaria, Pharmacology, Preventative medicine

## Abstract

**BACKGROUND:**

CIS43LS is a long-acting mAb that targets the *Plasmodium falciparum* circumsporozoite protein. A phase II trial showed that a single dose of CIS43LS conferred > 85% sterile protection against infection in Malian adults over 6 months. Understanding the pharmacokinetics and pharmacodynamics (PK/PD) of CIS43LS is critical for the further development of CIS43LS and other antimalaria mAbs.

**METHODS:**

Using 3,777 serum samples collected from 348 trial participants over the 6-month study period, we performed a PK/PD analysis of CIS43LS that included assessments for antidrug antibodies and target-mediated drug disposition. A 2-compartment, nonlinear mixed effects population PK model that evaluated demographic, anthropometric, hematologic, baseline parasitemia, and endogenous IgG and IgG1 as potential covariates was used to estimate PK parameters and serum concentrations required to achieve 80% efficacy.

**RESULTS:**

The median CIS43LS *t_1/2_* was 63.2 days (95% CI, 59.4–67.2 days). Serum concentrations ≥ 64 μg/mL (95% CI, 49–93 μg/mL) corresponded to ≥ 80% efficacy against infection over 6 months. A simulated dose of 30 mg/kg maintained serum concentrations > 64 μg/mL in > 97.5% of individuals for 4 months, the time frame for the WHO preferred product characteristics for antimalaria mAbs. There was no evidence of antidrug antibodies. Among infected individuals who received CIS43LS, no marked evidence of target-mediated drug disposition was observed.

**CONCLUSION:**

This study indicates that protective CIS43LS levels can be maintained over the course of a single malaria season and provides guidance for PK/PD analyses of antimalaria mAbs in malaria-endemic populations.

**TRIAL REGISTRATION:**

ClinicalTrials.gov NCT04329104.

**FUNDING:**

NIH and Gates Foundation.

## Abstract 2

Pharmacokinetic/pharmacodynamic analyses of the long-acting anti-malaria monoclonal antibody CIS43LS demonstrate that protective antibody concentrations can be maintained in malaria-exposed adults over a single malaria season.

## Introduction

Each year there are > 200 million cases of malaria resulting in over 600,000 deaths, mostly among children in Africa (1). Despite widespread deployment of insecticide-based interventions, early diagnosis and treatment, and chemoprevention in high-risk populations such as children and pregnant women, progress toward reducing malaria cases and deaths has stalled in recent years (1) and is further threatened by the emergence of insecticide-resistant mosquitoes (2) and drug-resistant parasites (3). WHO recently recommended the RTS,S/AS01 and R21/Matrix-M vaccines for malaria prevention in children aged 5 months to 3 years (1), in whom initial 3-dose regimens provide approximately 60%–75% efficacy against clinical malaria that can be partially maintained with booster doses (4, 5). Outside of this recommended age range, RTS,S/AS01 showed lower efficacy in younger infants (5) and adults in endemic areas (6). Thus, the development of new interventions that are safe and effective across all age groups and that complement existing countermeasures for high-risk populations (e.g., infants, pregnant women) is urgently needed.

CIS43LS (7), L9LS (8), and MAM01 (9) are long-acting mAbs in clinical development that target *Plasmodium* *falciparum* (*Pf*) sporozoites, the parasite stage that initiates infection in humans. These mAbs were engineered with the Met428Leu and Asn434Ser (LS) mutations in their Fc region, which enhances affinity to the neonatal Fc receptor (FcRn) to exploit FcRn-mediated antibody recycling, thus extending mAb *t_1/2_* (10). Antisporozoite mAbs administered intravenously or subcutaneously provide maximum serum concentrations within hours to several days and thus provide nearly immediate protection. Clinical studies with CIS43LS and L9LS have shown consistent protection across all ages evaluated to date (11). CIS43LS, which targets the conserved junctional region of the circumsporozoite protein (PfCSP) found on sporozoites (7), was the first antimalaria mAb to be evaluated in humans. A phase I trial involving US adults demonstrated the efficacy of CIS43LS against a single challenge with the bites of 5 *Pf*-infected mosquitoes (12). This was followed by a phase II trial of CIS43LS in Mali that involved adults exposed to intense, seasonal *Pf* transmission (13). In this trial, a single intravenous dose provided 75% and 88% protective efficacy against *Pf* infection at doses of 10 and 40 mg/kg, respectively, over a 6-month malaria season during which 87% of participants in the placebo group had *Pf* infections detected by RT-qPCR (13, 14). Similar efficacy was demonstrated against the appearance of blood gametocytes, the transmissible form of the parasite (14). Together, these data show that a single dose of an antisporozoite mAb can mediate sterile protection for 6 months to protect the host and potentially limit onward transmission. Determining the dose required to protect for a defined period through pharmacokinetic/pharmacodynamic (PK/PD) analyses is an essential step for the clinical development of mAbs.

The phase I trial of CIS43LS provided a preliminary PK/PD analysis in the context of a single controlled infection challenge in malaria-naive US adults (12, 15). It is critical to conduct larger PK/PD studies of antimalaria mAbs in endemic areas where individuals are exposed to repeated *Pf* infections over time to delineate dosing regimens that maximize efficacy and duration of protection while minimizing cost — a critical step toward equitable access to mAbs in low-income countries (16). In addition, it is important to identify factors in endemic areas that might influence the PK/PD of antimalaria mAbs. For example, it is unknown whether repeated *Pf* infections accelerate mAb clearance through target-mediated drug disposition (TMDD) (17) or whether the risk of developing anti-drug antibodies (ADAs) differs in malaria-exposed populations, which would have important implications for clinical use cases that may require repeated dosing, such as seasonal malaria prevention in children or malaria prevention over multiple pregnancies. Here, we report a comprehensive PK/PD analysis of CIS43LS that includes assessments of ADAs and TMDD in participants of an antimalaria mAb efficacy trial conducted in an area where malaria is endemic.

## Results

### Study design, participants, and efficacy results.

**Figure 1:**
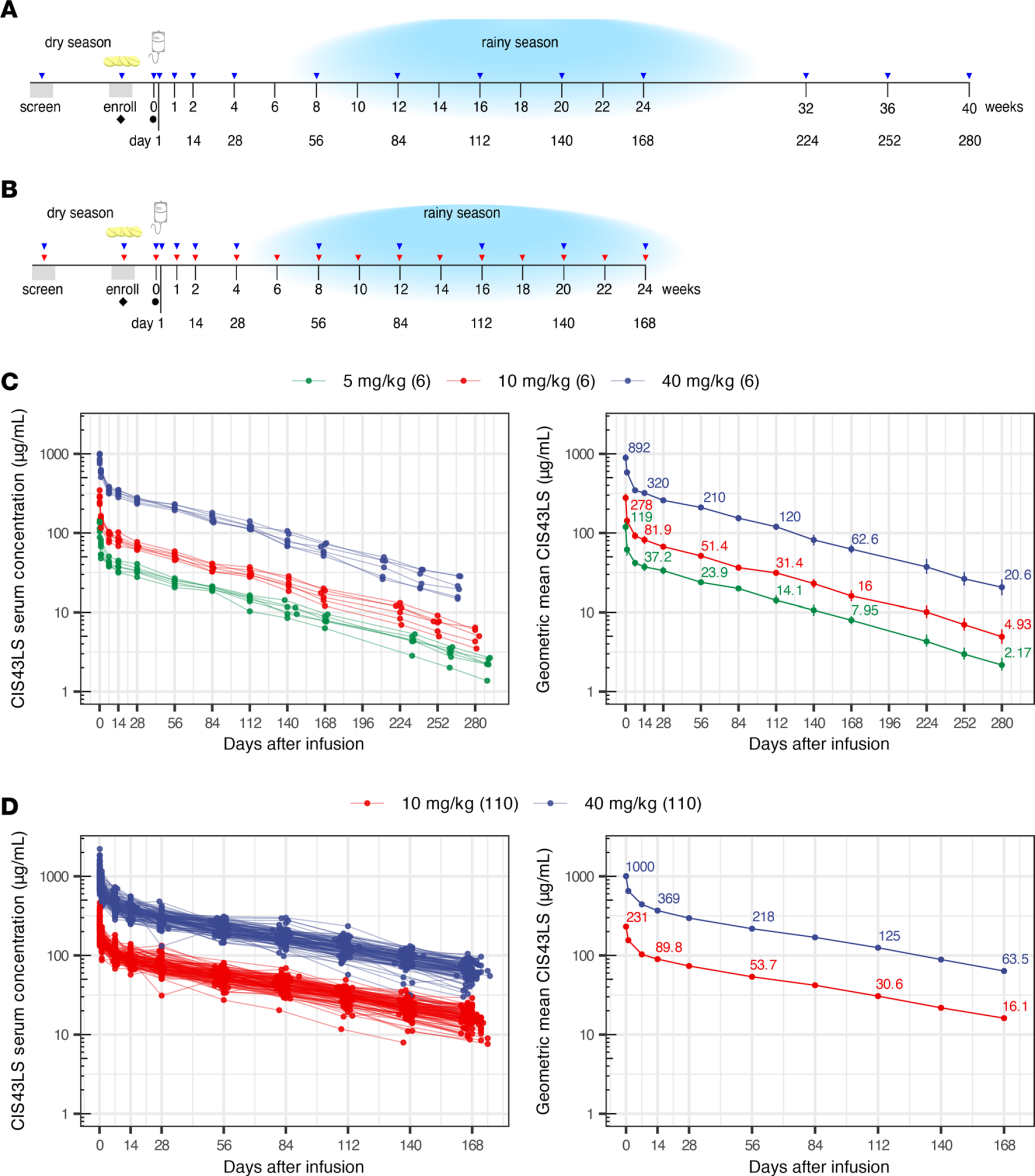
CIS43LS serum concentration-time profiles in Malian adults. (**A** and **B**) Design of the dose escalation (**A**) and efficacy (**B**) studies. Black diamond represents a 3-day course of artemether-lumefantrine initiated at enrollment 7–15 days (median 9 days) before CIS43LS or placebo administration on day 0. Black circle represents day of infusion of 10 mg/kg of CIS43LS (*n* = 110), 40 mg/kg of CIS43LS (*n* = 110), or placebo (*n* = 110). Red inverted triangles represent thick blood smear collections for the detection of *Pf* parasites, and blue inverted triangles represent serum collections for PK analysis. On day 0, serum was collected before and 1 hour after CIS43LS or placebo administration. (**C** and **D**) Observed and geometric mean CIS43LS serum concentrations for participants enrolled in the dose escalation (**C**) and efficacy (**D**) studies. For the left panels in **C** and **D**, lines connect longitudinally collected observations from individual participants. For the right panels in **C** and **D**, error bars represent 95% bootstrap CIs around the geometric mean. Group sample sizes are shown in parentheses.

As previously reported, we conducted a 2-part, phase II trial in Mali to assess the safety, tolerability, and efficacy of a single intravenous infusion of CIS43LS against *Pf* infection in healthy adults during the 6-month rainy season when malaria transmission occurs (13). An open-label, age deescalation and dose escalation study was first conducted during the dry season to assess the safety, tolerability, and PK of 3 escalating dose levels of CIS43LS in 18 adults: 5 mg/kg (*n* = 6), 10 mg/kg (*n* = 6), and 40 mg/kg (*n* = 6) (Figure 1A). This was followed by an efficacy study initiated before the rainy season (Figure 1B) in which 330 adults were randomly assigned in a double-blind manner to receive 10 mg/kg of CIS43LS (*n* = 110), 40 mg/kg of CIS43LS (*n* = 110), or placebo (*n* = 110). The primary efficacy endpoint, assessed in a time-to-event analysis, was the first *Pf* infection detected on thick blood smear examination with an onset between weeks 1 and 24 after administration of CIS43LS or placebo. Blood smear examinations were performed at least every 2 weeks for 24 weeks during the rainy season. At enrollment, 7–15 days (median 9 days) before CIS43LS or placebo administration, all participants received a 3-day course of artemether-lumefantrine to clear possible *Pf* infection.

As previously reported, all participants in the dose escalation study and 311 of 330 participants (94.2%) in the efficacy study completed follow-up through the last scheduled study visit, and there were no evident safety concerns (13). Baseline characteristics among participants in the efficacy study were similar across study arms (13). Among the 330 efficacy study participants included in the modified intention-to-treat dataset (i.e., those who had undergone randomization and received CIS43LS or placebo), *Pf* infections were detected by thick blood smear over the 6-month study period in 78.2% of participants who received placebo, 35.5% of participants who received 10 mg/kg of CIS43LS, and 18.2% who received 40 mg/kg of CIS43LS. At 6 months, in time-to-event analysis, the efficacy of 40 mg/kg of CIS43LS compared with placebo was 88.2% (adjusted 95% CI, 79.3–93.3; *P* < 0.001), and the efficacy of 10 mg/kg of CIS43LS compared with placebo was 75.0% (adjusted 95% CI, 61.0–84.0; *P* < 0.001) (13).

### CIS43LS PK.

CIS43LS PK was evaluated for all study participants. For the 18 participants in the dose escalation study, serum was collected for PK analysis at 14 prespecified time points before and up to 40 weeks after CIS43LS administration (Figure 1A). Of these 252 serum collections, 251 (99.6%) were available and analyzed. For the 330 participants in the efficacy study, serum was collected for PK analysis at 11 prespecified time points before and up to 24 weeks after CIS43LS or placebo administration (Figure 1B). Of these 3,630 serum collections, 3,526 (97.1%) were available and analyzed. Serum concentrations of CIS43LS were measured on the Meso Scale Diagnostics (MSD) Discovery platform using a CIS43 anti-idiotype antibody by investigators who were blinded to study arm assignments.

In both the dose escalation and efficacy studies, the PK profile of CIS43LS was dose linear and biphasic, showing a rapid distribution followed by slower, linear elimination with persistent serum concentrations over the study period (Figure 1, C and D). Given that PK profiles were similar for the 10 and 40 mg/kg dose groups across the 2 studies, dose groups were combined across both studies to assess CIS43LS exposure parameters (Supplemental Table 1; supplemental material available online with this article; https://doi.org/10.1172/JCI207559DS1). The maximum serum concentration (C_max_) occurred during the immediate postinfusion period (measured ~1 hour after infusion; Figure 1, C and D, and Supplemental Table 1). Evaluation of mean serum C_max_, concentrations at day 168 (C_168d_) and day 252 (C_252d_), and the area under the concentration–time curve from infusion to day 168 (AUC_0-168d_) confirmed dose linearity across the 5–40 mg/kg dosing range for the duration of the study period (Supplemental Table 1).

### Malaria exposure and CIS43LS PK.

**Figure 2:**
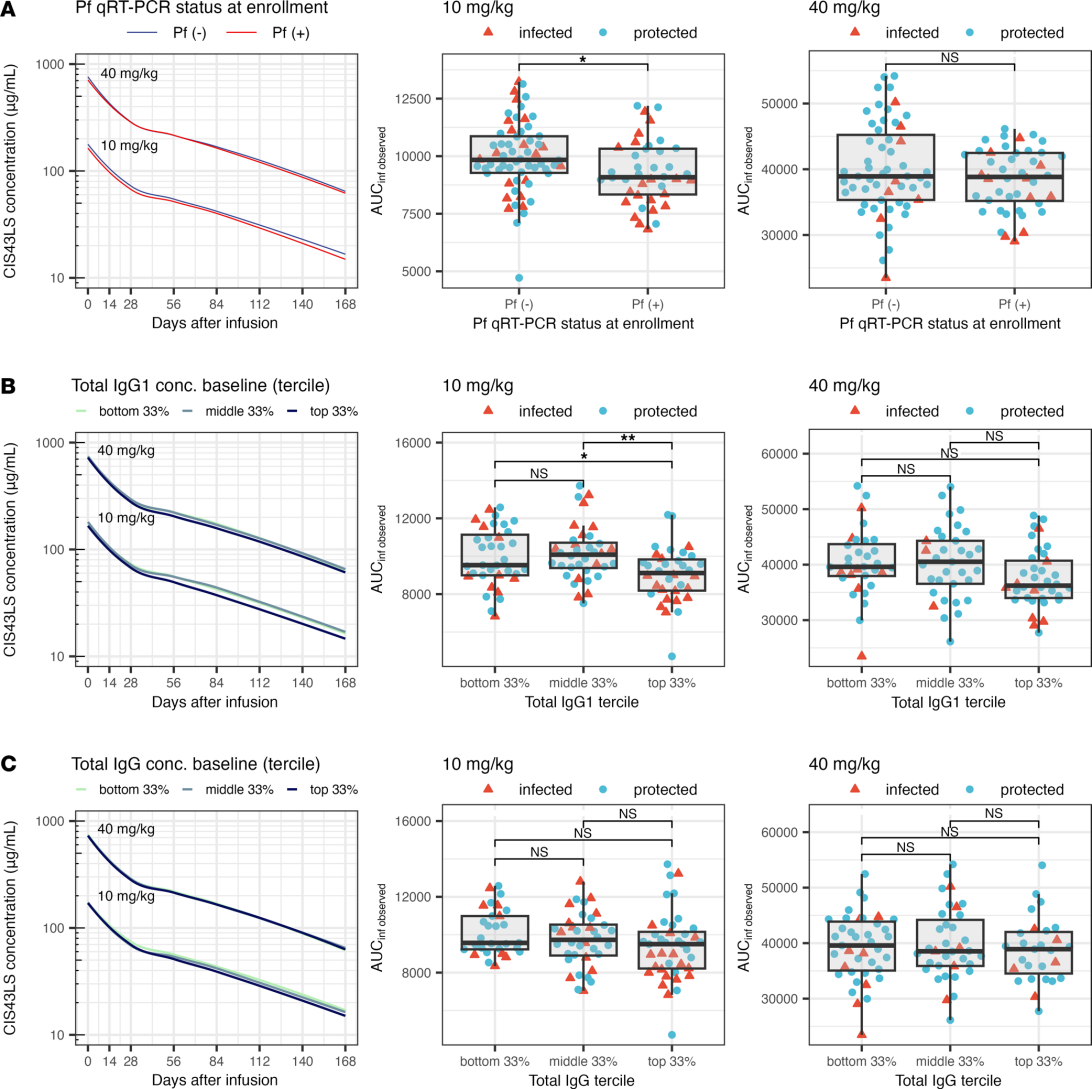
Effect of *Pf* infection status at enrollment and serum IgG/IgG1 levels on CIS43LS clearance and exposure. Loess fit curves of observed concentrations versus time and AUC_inf_ _observed_ (from 0 to infinity, extrapolated from the last observed concentrations) by *Pf* RT-qPCR status at enrollment (**A**), baseline serum IgG1 terciles (**B**), and baseline serum IgG terciles (**C**) for each dose group. Individuals who became infected with *Pf* by blood smear during the surveillance period are denoted by red triangles. Significance was determined by 1-way ANOVA with Tukey’s 2-sided post hoc test. *P < 0.05, **P < 0.01.

In post hoc analyses, we explored whether variables related to malaria exposure (henceforth referred to as malariometric indices) affected CIS43LS PK. We first compared (a) loess fit curves of CIS43LS concentration versus time and (b) CIS43LS drug exposure (estimated by AUC) grouped by *Pf* RT-qPCR positivity at enrollment as a surrogate for individual-level *Pf* infection risk. AUC values for participants who were *Pf* positive at enrollment were significantly lower than those who were *Pf* negative in the 10 mg/kg but not the 40 mg/kg dose group, albeit with a small effect size (Figure 2A). Second, we examined the relationship between CIS43LS serum concentration and endogenous serum IgG levels, since malaria exposure is associated with an approximately 1.3-fold increase in serum IgG levels at the population level, as evidenced by higher IgG and IgG1 levels in Africans living in malaria-endemic countries compared with Africans living in Europe (18) or non-Africans (19). Higher endogenous serum IgG can potentially increase mAb clearance by saturating the FcRn-mediated IgG recycling pathway, with IgG1, being the dominant subclass, having the largest overall effect (20, 21). The top tercile of serum concentrations of total (nonspecific) IgG1, but not total IgG, was associated with faster CIS43LS clearance and lower AUCs, a finding that was more pronounced and significant in the 10 mg/kg group (Figure 2, B and C). Notably, among 10 mg/kg CIS43LS recipients who were *Pf* positive at enrollment or had the highest baseline IgG1 concentrations, those who acquired new *Pf* infections during the surveillance period had significantly lower CIS43LS AUCs compared with those who did not (Figure 2, A and B, and Supplemental Table 2).

### Evaluating CIS43LS PK among participants who acquired new Pf infections.

**Figure 3:**
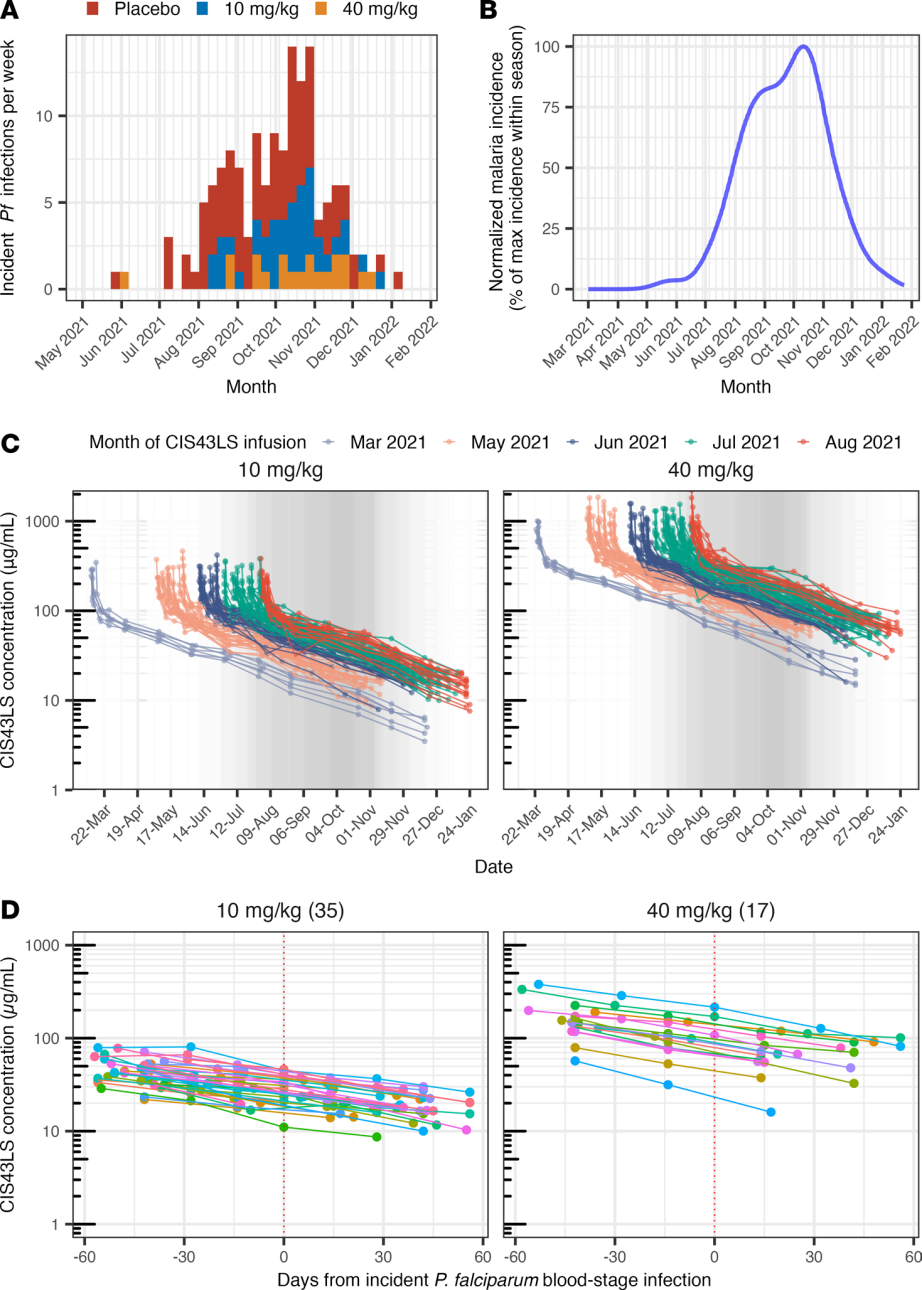
Relationship between CIS43LS PK and malaria exposure. (**A**) Histogram of first detected *Pf* infections by blood smear binned by 7-day periods and separated by study arm for the efficacy study conducted from May 2021 to February 2022. (**B**) Kernel density estimates for incident *Pf* infections in the placebo group from **A**, normalized as a percentage of the maximum daily incidence during the malaria season. (**C**) Observed concentrations versus time by month of CIS43LS infusion at 10 and 40 mg/kg doses. Longitudinal observations for each participant are shown by connecting lines. Gray background intensity represents normalized malaria incidence in **B**. (**D**) Observed concentrations centered around the time of incident parasitemia for participants who were infected at ≥ 28 days after infusion by dose group (sample size). Color represents longitudinal observations for a participant. Vertical dotted line is the time of first positive smear.

A key aspect of this study was to evaluate the relationship between *Pf* exposure and CIS43LS PK over the course of the 6-month malaria season during which malaria transmission intensity peaks in October (22) (Figure 3, A and B). CIS43LS serum concentration versus calendar time of CIS43LS infusion showed minor interval deviations from linearity for some participants during the terminal elimination phase (≥28 days after infusion), particularly at times approaching the peak of malaria transmission (Figure 3C). We investigated whether *Pf* infections per se contributed to these deviations by examining PK profiles of participants who had incident, RT-qPCR–confirmed *Pf* infections during the terminal elimination phase of CIS43LS. We found that linear elimination of CIS43LS was consistently maintained during the 120-day interval spanning the time of incident *Pf* infection (Figure 3D), suggesting that *Pf* infections did not contribute to the observed deviations. This is consistent with the hypothesis that TMDD would not significantly impact CIS43LS PK since *Pf*-infected mosquitoes inoculate at most 10^4^ sporozoites into human skin and blood (23), and PfCSP is not expressed during the subsequent erythrocytic stage (24). In contrast to the sporozoite stage of infection, the erythrocytic stage typically generate up to 10^10^ parasites during acute malaria (25), so we also assessed off-target binding of CIS43 (without the LS mutation) to erythrocytic stage antigens using a protein microarray containing 8,873 *Pf* full-length or fragmented proteins representing 5,233 unique genes from the Pf3D7 genome (~99% of the Pf3D7 protein-coding genome). Consistent with our observation that *Pf* infections were not associated with deviations in CIS43LS PK, we found that CIS43 bound to its cognate antigen PfCSP and to only 5 other antigens expressed during the erythrocytic stage, none of which are known to be cell-surface proteins (Supplemental Figure 1). Taken together, these data suggest that *Pf* infections do not substantially impact CIS43LS PK, even in a high transmission setting, and that other factors associated with the malaria season might underlie the observed minor PK deviations.

### CIS43LS PD and protective efficacy.

**Figure 4:**
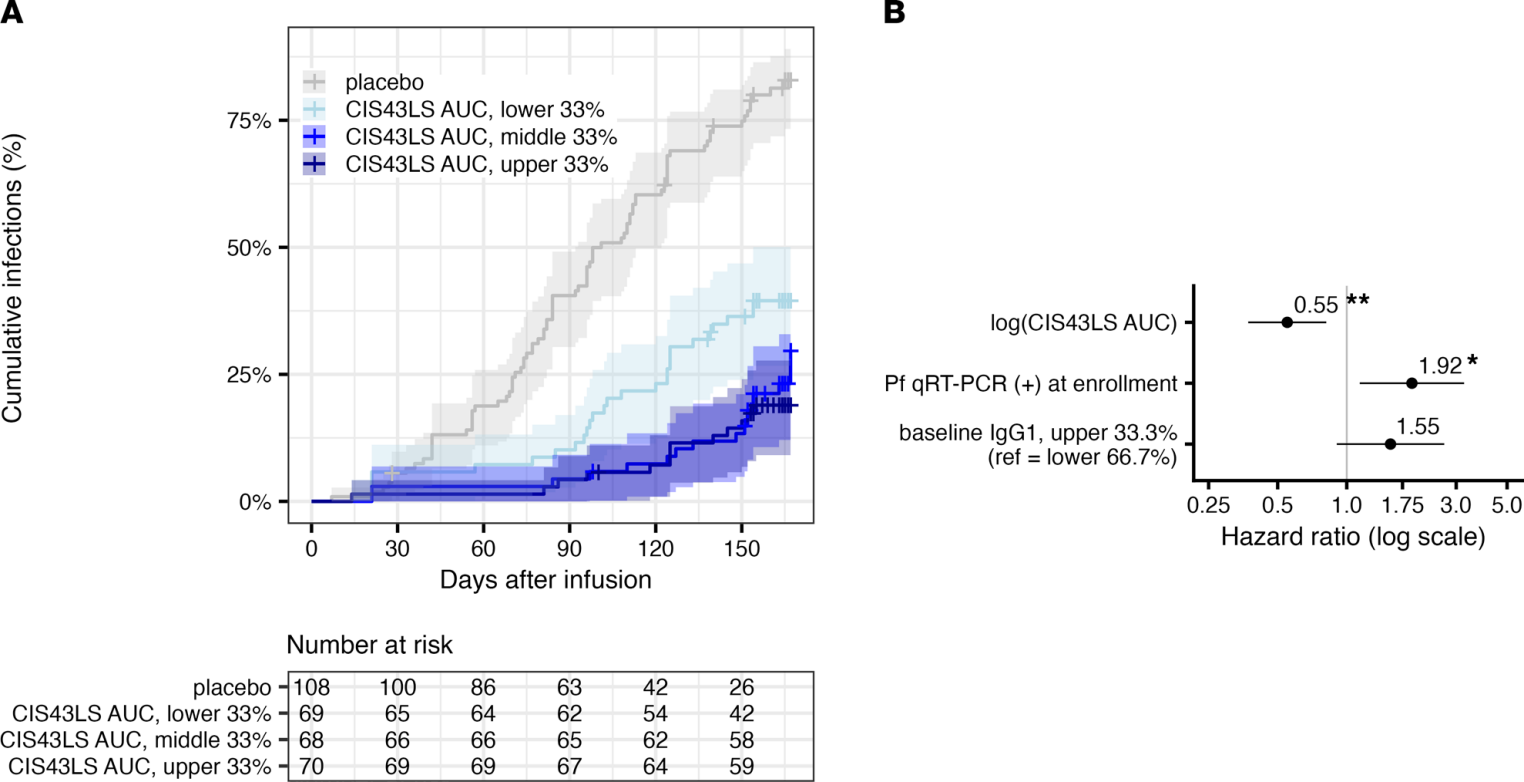
Total CIS43LS exposure and risk of *Pf* infection. (**A**) Cumulative hazard for each area under the serum CIS43LS concentration-time curve (AUC) tercile. Terciles were determined from AUC_0-140d_ to allow inclusion of participants whose last observed CIS43LS concentration occurred just prior to 168 days. (**B**) Hazard ratios with 95% CIs for a Cox proportional hazards model with CIS43LS AUC as a time-varying covariate and *Pf* positivity by RT-qPCR at enrollment and baseline IgG1 concentration terciles as time-invariant covariates. The analysis was limited to the 202 CIS43LS recipients in the efficacy study for whom both AUC and enrollment *Pf* RT-qPCR data were available. Significance was determined by 2-sided Wald’s test on 3 degrees of freedom. *P < 0.05, **P < 0.01.

To further assess PK variability and efficacy, a CIS43LS exposure-response analysis was performed for 219 of the 220 participants who received CIS43LS in the efficacy study. Higher total CIS43LS serum concentration during the surveillance period, estimated as AUC terciles, was significantly associated with reduced infection risk (Figure 4A and Supplemental Table 3). This association remained significant when AUC was treated as a time-varying continuous variable and after controlling for (a) baseline *Pf* infection as detected by RT-qPCR, which has been shown to predict increased *Pf* reinfection risk in prospective analyses (22, 26) and thus estimates individual-level *Pf* risk, and (b) baseline serum IgG1 concentrations (Figure 4B). As expected, individuals infected with *Pf* at enrollment had a significantly higher risk of subsequent *Pf* infection. High baseline total IgG1 serum concentrations in the upper tercile were associated with increased risk of incident *Pf* infection, although this finding was not statistically significant.

**Figure 5:**
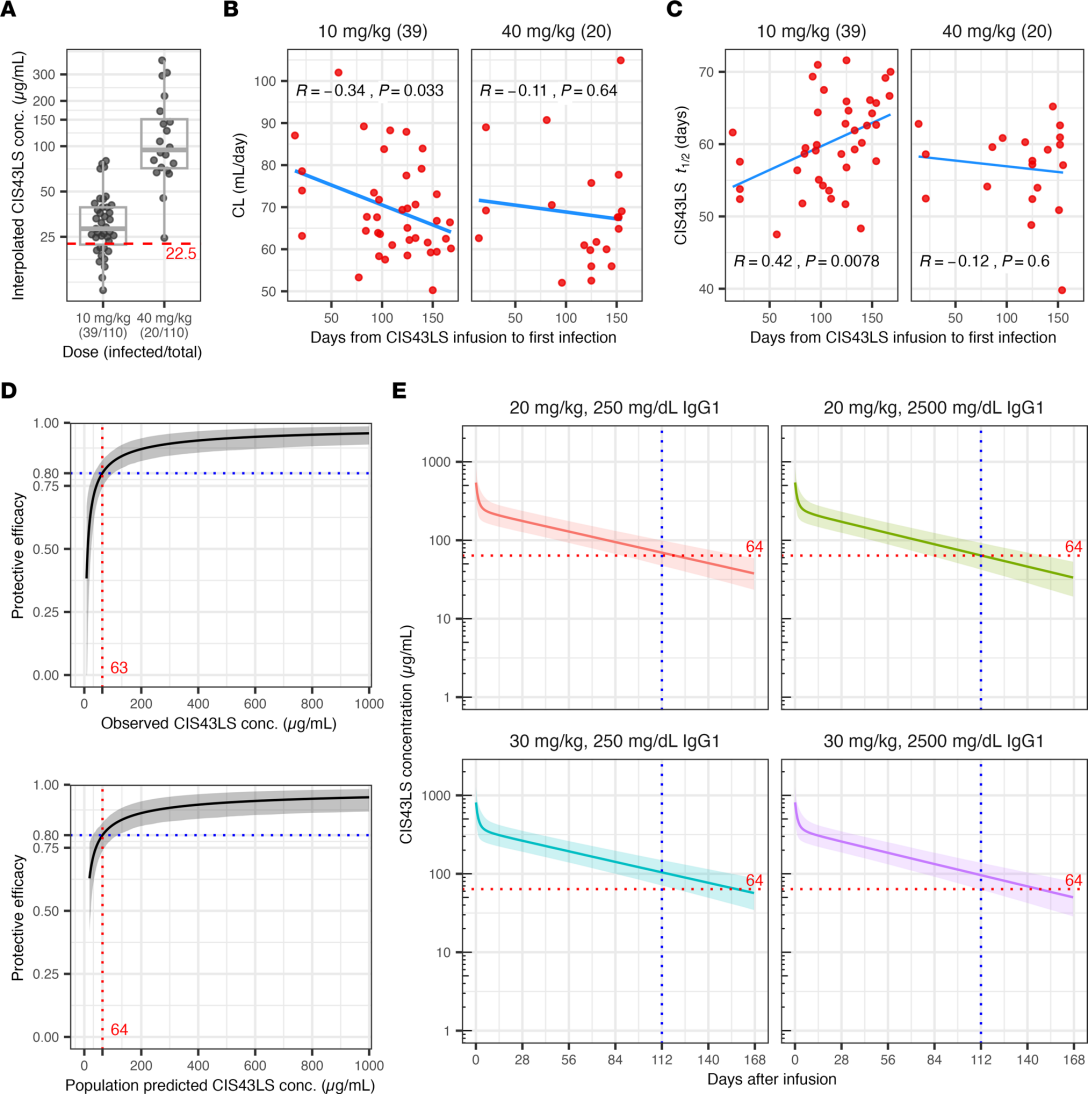
CIS43LS PD in the Malian efficacy trial. (**A**) CIS43LS concentration 7 days prior to first positive blood smear in infected individuals by dose group. Values were interpolated from observed concentrations. Dashed line represents concentration estimated to provide 90% protective efficacy in VRC 612 Part C. (**B** and **C**) Pearson’s correlation between days from CIS43LS infusion to first positive blood smear and CL (**B**) or *t_1/2β_* (**C**) by dose group. 2-sided *P* values are shown. (**D**) Concentration versus efficacy plot showing the minimum CIS43LS serum concentration required to protect against natural *Pf* infections in the context of intense seasonal transmission at 80% efficacy. Analysis was performed by Cox regression with the randomization arm (CIS43LS or placebo, time-fixed) and CIS43LS concentration (time-varying) as regressors, combining both dose arms. Top and bottom plots used CIS43LS concentrations interpolated from observed values or predicted from the PopPK model, respectively. Shown are bootstrapped 95% confidence bands (1,000 simulations). (**E**) PK simulations of cohorts (220 individuals/cohort) with baseline serum IgG1 concentrations of 250 or 2,500 mg/dL dosed at either 20 or 30 mg/kg CIS43LS using the PopPK model derived from the Malian efficacy trial. For each simulated condition, the curve represents the median concentration with 2.5 and 97.5 percentile bands of 1,000 simulations. Red and blue dotted lines represent CIS43LS concentration required for 80% efficacy determined in **D** and the WHO preferred duration for antimalarial mAb efficacy of approximately 4 months, respectively.

We next estimated the concentration required to mediate a defined level of protective efficacy. In this Mali efficacy trial, 83% of incident *Pf* infections among CIS43LS recipients occurred at CIS43LS concentrations above 22.5 μg/mL (Figure 5A), the threshold estimated to provide 90% protective efficacy against a single controlled *Pf* infection in the phase I trial involving North American adults (VRC 612 Part C) (15). Notably, in the Mali trial, 9 of 20 infected participants in the 40 mg/kg group were infected early during the study, when serum CIS43LS concentrations were above 100 μg/mL (Figure 5A and Supplemental Figure 2). This implies that CIS43LS concentrations much higher than 22.5 μg/mL are needed to achieve > 80% protective efficacy in endemic areas where populations are repeatedly exposed to naturally occurring *Pf* infections. To better estimate CIS43LS PK parameters and thus predict CIS43LS concentrations in individuals exposed to seasonal malaria transmission, we developed a 2-compartment, nonlinear mixed effects population PK model (PopPK) by evaluating demographic, anthropometric, hematologic, and malariometric indices available in the Mali efficacy trial as potential covariates, with allometric scaling based on a standard body weight of 70 kg (Supplemental Table 4 and Supplemental Figure 3). The final model included baseline serum IgG1, age, and allometric scaling as covariates on the clearance (CL) parameter and dose, sex, and allometric scaling as covariates on the central volume (Vc) parameter (Table 1). Overall, simulations from this PopPK model reliably reproduced the 10th, 50th, and 90th percentiles during the elimination phase for both dose groups, with slight underprediction of lower, 10th percentile values at days ≥ 84 days (3 months) for the 40 mg/kg dose group (Supplemental Figure 4). The elimination half-life (*t*_1/2β_) estimated by the 2-compartment model was 63.2 (95% CI, 59.4–67.2) days. Clearance was estimated as 32.1 (95% CI, 30.5–33.9) mL/day. The volume of distribution at steady state (Vss) was estimated to be 2.93 (95% CI, 2.84–3.02) L. Baseline serum IgG1 had a positive effect (0.033; 95% CI, 0.00014–0.073) on CL, whereas age had a negative effect on CL (–0.0022; 95% CI, –0.0038 to –0.00064). Both dose and female sex had negative effects on Vc (Table 1).

Among infected participants who received the lower 10 mg/kg dose, days to first infection negatively correlated with CIS43LS CL and, conversely, positively correlated with *t*_1/2β_ (Figure 5, B and C). Similar significant correlations were not observed in the 40 mg/kg group, suggesting that higher doses of CIS43LS extend protection among those who eliminate CIS43LS at a faster rate. Using concentrations either interpolated from observed values or predicted from the PopPK model, we estimated that CIS43LS concentrations of 63 (95% CI, 34–102) μg/mL or 64 (95% CI, 30–108) μg/mL would provide ≥ 80% protective efficacy against *Pf* infection for 6 months in this malaria-endemic adult population (Figure 5D). The WHO preferred product characteristics for antimalaria mAbs include ≥ 80% preventive efficacy against clinical disease for 3–4 months (27). In simulated cohorts, a single 30 mg/kg dose of CIS43LS would maintain serum concentrations above the protective threshold of 64 μg/mL in > 97.5% of individuals for 112 days (3–4 months), even when accounting for high endogenous IgG1 concentrations present in some Malian adults (Figure 5E and Supplemental Table 4). Notably, the CIS43LS dose required to provide ≥ 70% protective efficacy for 112 days in > 97.5% of individuals was estimated to be 12.5 mg/kg, as CIS43LS serum concentrations would only need to be maintained above a 28 (95% CI, 18–49) μg/mL threshold (Supplemental Figure 5).

### ADAs.

ADAs can affect the PK, efficacy, and safety of mAbs. We used a 2-tier assay to detect ADAs in serum collected from participants in both the dose escalation and efficacy studies (*n* = 238 across all CIS43LS dosing groups and *n* = 110 for placebo). Serum was assayed at day 0 (before administration of CIS43LS or placebo) and at days 7, 28, 84, and 168 thereafter. The tier 1 assay is a standard bridging assay intended for screening, and the tier 2 assay is a confirmatory screen with competition from unlabeled antibody to determine specificity (28). Of the 1,740 serum collections prespecified for ADA assessment, 1,708 (98.2%) were available and assayed. Of these, 154 samples (9.0%) were positive for ADAs in the tier 1 screen (Supplemental Figure 6A). Of these, only 3 samples (0.18%) had detectable ADAs in the confirmatory screen: 2 participants had ADAs detected on day 0 prior to receiving CIS43LS, and no ADAs were detected at subsequent time points in these 2 participants (Supplemental Figure 6, B and C). The third participant received 10 mg/kg of CIS43LS and had low-level ADAs detected (21.8% inhibition; positive cutoff 19.4%) on day 84 but was negative at all other time points (Supplemental Figure 6C).

## Discussion

Here, we report a comprehensive PK/PD analysis of an antimalaria mAb with the *t_1/2_* extending LS mutation (29, 30) in an efficacy trial of individuals living in a malaria-endemic region of sub–Saharan Africa, building upon work that evaluated the PK of broadly neutralizing LS mAbs for HIV prevention in phase I studies of women in South Africa (31, 32). In the present trial, the availability of frequent serum sampling as well as hematological and malariometric indices allowed us to evaluate whether these factors impacted the distribution and clearance of CIS43LS and, subsequently, the risk of *Pf* infection. Of the malariometric indices, we found that having either *Pf* parasitemia or high endogenous IgG1 concentrations at baseline was associated with lower CIS43LS drug exposure (as measured by AUC), but only in the lower 10 mg/kg dosing group. This suggests that the negative effects of parasitemia and endogenous IgG1 on CIS43LS exposure can be overcome by increasing the dose of the mAb.

Of the hematological and malariometric indices, only endogenous total IgG1 levels contributed to increased clearance when formally evaluated in a PopPK model. Although a small effect, this finding is consistent with the concentration-catabolism effect, whereby high serum IgG levels saturate the FcRn-mediated recycling pathway, limiting its own rescue from lysosomal degradation and thus leading to faster clearance (33, 34). This phenomenon can occur whether the excess IgG is endogenous (35, 36) or exogenously administered (37) and can also affect the clearance of therapeutic mAbs (21, 38), which are primarily IgG1. Repeated *Pf* infections can induce nonspecific polyclonal hypergammaglobulinemia (39, 40), which has been hypothesized to be the basis for higher total serum IgG in malaria-endemic populations relative to nonendemic populations (18, 19, 41, 42). The significant effect of higher endogenous IgG1, but not IgG, on CIS43LS elimination observed here may be attributable to IgG1 having higher affinity for FcRn relative to IgG, which consists of all subclasses, including IgG3, which has lower affinity for FcRn (43, 44).

The *t*_1/2β_ of 63 days for CIS43LS estimated here in Malian adults is shorter than the 80 days estimated for CIS43LS in the phase I study of malaria-naive North American adults (15). It is unlikely that TMDD is responsible for the shorter CIS43LS *t_1/2_* observed in the Mali trial, as CIS43LS PK was not markedly impacted by exposure to its target antigen PfCSP via *Pf* infections. ADAs were also not responsible for the shorter *t_1/2_* of CIS43LS. The small effect of endogenous serum IgG1 on increased clearance observed in the Mali study may not fully explain the disparity in CIS43LS half-lives, since clearance estimates were similar for Malian (32.2 mL/day) and North American (33.7 mL/day) adults, despite lower endogenous IgG1 in the latter (data not shown). The shorter *t_1/2_* observed in Malians may be attributable to reduced steady-state distribution volumes among Malians (Vss; 2.93 L) relative to those of North Americans (3.79 L). Unknown genetic or environmental factors (e.g., pathogen exposures) that affect convective extravasation, FcRn-mediated transcytosis of mAbs, or distributional properties unique to LS mAbs may further contribute to differences in Vss between Malian and North American adults (45–47). Lower extracellular water fraction (which directly correlates with volume of distribution) could also contribute to lower Vss in Malians relative to North Americans (48, 49). Notably, the geographic differences in CIS43LS PK features observed here are consistent with the only published study that directly compared mAb PK parameters between African and non-African cohorts. In phase IIb trials of the HIV-1–neutralizing mAb VRC01 (Antibody Mediated Prevention trials), the *t*_1/2β_ and Vss for VRC01 were significantly lower in sub–Saharan African women compared with predominantly non-black men and transgender persons from the Americas and Switzerland, after adjusting for dose, age, race, weight, and creatinine clearance (50).

In the present study, the CIS43LS serum concentration that corresponded to ≥ 80% protective efficacy was 64 μg/mL. This is considerably less than the effective serum concentration of 364 μg/mL of CIS43LS needed for 80% protection (EC_80_) (7, 8) in preclinical models, whereby mice were challenged once by bites of mosquitoes infected with chimeric *Plasmodium berghei* parasites containing PfCSP. In contrast, 22.5 μg/mL was the minimum CIS43LS concentration needed for 90% protection from *Pf* infection after a single controlled infection challenge with 5 *Pf*-infected mosquito bites in malaria-naive North American adults (VRC 612) (15). Thus, mouse models and controlled infection studies of healthy adults may overestimate and underestimate, respectively, the anti-PfCSP mAb concentrations required for protection in malaria-endemic settings. The higher protective CIS43LS concentration threshold observed in the current study relative to the North American study may be due to the intense malaria transmission at the study site in Mali, where individuals can experience over 50 genetically distinct *Pf* infections during a single 6-month malaria season (51). In this setting, occasional *Pf* infections may still occur even when CIS43LS serum concentrations are well above the protective threshold as only a single sporozoite is required to initiate blood stage infection. Potential explanations for the observed breakthrough infections occurring at high CIS43LS serum concentrations include preexisting, naturally acquired anti-PfCSP antibodies and/or genetic polymorphisms within PfCSP that reduce the ability of CIS43LS to bind the junctional epitope and thus neutralize sporozoites. Both possibilities are currently being investigated. Moreover, ongoing PK/PD analyses of trials in Africa of L9LS, another PfCSP-specific mAb in clinical development that was more potent than CIS43LS in mouse models (8, 26), will determine whether the relative differences in EC_80_ across mouse and human studies are consistent, which would help establish reliable benchmarks for prioritizing the clinical development of candidate anti-PfCSP mAbs.

Modeling results from this study indicate that a single 30 mg/kg i.v. dose of CIS43LS would maintain serum concentrations above the 64 μg/mL threshold needed for ≥ 80% protective efficacy against *Pf* infection for 3–4 months in > 97.5% of adults exposed to intense, seasonal malaria transmission, thus meeting the WHO preferred product characteristics for efficacy and duration of protection for antimalaria mAbs (27). Of note, ongoing trials of L9LS are assessing a dose of approximately 30 mg/kg in infants and children, either as a fixed dose of 150 mg administered intramuscularly to healthy infants in Mali (ClinicalTrials.gov NCT06461026) or as a 30 mg/kg i.v. dose administered to children in Kenya with severe anemia or severe malaria to prevent malaria after hospital discharge (ClinicalTrials.gov NCT07082205). Importantly, in the present study, a CIS43LS dose of only 12.5 mg/kg is predicted to maintain serum concentrations above a threshold of 28 μg/mL needed for ≥ 70% protective efficacy for 3–4 months. It is likely that this level of protection would have meaningful public health benefits across different use cases and have implications for improving the cost-effectiveness of antimalaria mAbs. To provide context for using mAbs to prevent other infections, prior to the spread of the SARS-CoV-2 Omicron variant, casirivimab/imdevimab, when used for prophylaxis at doses of 600 mg each (1,200 mg total or 18 mg/kg for the average adult), could maintain plasma concentrations above a 0.40 μg/mL threshold estimated to provide 50% protection against COVID-19 disease for approximately 8 months (52). The higher protective CIS43LS concentration threshold for CIS43LS may be due to the intense nature of *Pf* transmission at the study site (versus episodic SARS-CoV-2 exposure) as well as the infection endpoint used in the CIS43LS trial (versus a disease endpoint in the SARS-CoV-2 study).

The current study provides data on the PK/PD of an intravenously administered antimalaria mAb in an adult population exposed to intense seasonal malaria transmission. However, given known differences in the distribution and clearance of mAbs in adults and children (53), the findings of this study may not be generalizable to children < 5 years of age, the population at highest risk of severe and fatal malaria that would benefit most from antimalaria mAbs. Indeed, the *t_1/2_* of nirsevimab, an anti–respiratory syncytial virus mAb with an extended *t_1/2_*, appears to be shorter in infants than adults (54, 55). Thus, it is important to conduct PK/PD analyses of antimalaria mAb trials that involve infants and children residing in different malaria transmission settings. Similar PK/PD analyses are being applied to trials of the anti-PfCSP mAb L9LS that involve infants and children in Mali (ClinicalTrials.gov NCT05304611 and NCT06461026) (26) and Kenya (ClinicalTrials.gov NCT05400655 and NCT07082205) (56).

Importantly, no ADAs were detected after a single i.v. dose of CIS43LS in this study. Additional studies are needed to assess whether ADAs develop after repeated doses of antimalaria mAbs or when administered by subcutaneous or intramuscular injection, both of which may increase the risk of ADAs (57). Accordingly, ADA risk is being assessed in the trials of L9LS in Malian (ClinicalTrials.gov NCT05304611) (26) and Kenyan (ClinicalTrials.gov NCT05400655) children in which 2 doses of L9LS are given subcutaneously at least 6 months apart (56).

There are limitations to this study. As noted above, the PK parameters estimated using data from a malaria-endemic adult population may not be applicable to children living in malaria-endemic communities given age-related differences in weight-normalized mAb distribution volumes and clearance secondary to physiological differences in mAb extravasation, FcRn-mediated recycling, and tissue maturation (53). In addition, we used detectable *Pf* blood-stage infections as a proxy for sporozoite exposure when assessing for evidence of TMDD, which likely underestimates the true number of sporozoite exposure events. Lastly, our study does not account for genetic polymorphisms in FcRn or endogenous IgG1 allotypes, both of which could affect CIS43LS PK (20, 58).

In summary, this study demonstrates that protective serum concentrations of CIS43LS can be predictably maintained for defined intervals in a population residing in a malaria-endemic area that is exposed to repeated *Pf* infections. These results support the continued development of antimalaria mAbs for use in populations at high risk of malaria for defined periods and provide important guidance for the planning and analysis of future trials of antimalaria mAbs.

## Methods

### Sex as a biological variable

The CIS43LS clinical trial enrolled both male and female adults in a randomized manner. Sex was formally evaluated as a covariate on the PK parameters in the PopPK model, as noted in “Statistics.”

### Automated PK measurements

CIS43LS anti-idiotype (ID) 1-1 antibody was spot coated at 125 μg/mL on 384-well standard bind plates and packaged by MSD. Sample and reagent handling was performed on a Beckman Biomek i7 automated workstation in accordance with the assay approach described by Kayentao et al. (26). Briefly, plates were blocked for 1 hour with MSD Blocker A solution. Test samples and standard were serially diluted in assay diluent (MSD Diluent 100). Blocked plates were washed, and diluted test samples, standards, and controls were added to the washed assay plates. Plates were incubated with shaking for 4 hours at room temperature. Plates were washed with Sulfo-tag–labeled mouse anti-human IgG detection antibody in assay diluent, and 1.5 μg/mL was applied to the plates and allowed to associate with complexed anti-ID/CIS43LS within the assay wells for 1 hour with shaking. The plates were washed to remove unbound detection antibody, and MSD Read Buffer containing Sulfo-Tag substrate was immediately added to the wells. Plates were read using the MSD Sector Imager S600. As current was applied to the plate, areas of well surface that formed a full anti-ID/CIS43LS/Sulfo-Tag anti-human IgG complex emitted light in an electrochemiluminescence (ECL) reaction. The amount of CIS43LS sandwiched by the anti-ID and anti-human IgG antibodies was directly proportional to the concentration of reactive CIS43LS. Serial dilutions of sample within the dynamic range of the standard curve were interpolated to assign a sample concentration. Analysis was performed using MSD Discovery Workbench Software and Microsoft Excel. The qualified quantitation range of the assay was 666 ng/mL to 5,122 μg/mL.

### Automated ADA detection

A 2-tiered approach was used to screen and confirm ADAs in clinical serum samples on a Beckman Biomek FX automated workstation. For the tier 1 screening assay, sera of the participants were prediluted at 1:2 in assay diluent (1% MSD blocking buffer and 0.05% Tween in 1× PBS) and mixed at 1:1 with an optimized concentration of Sulfo-tag–labeled CIS43LS (reporter molecule) and biotinylated CIS43LS (capture molecule) for a final sample dilution of 1:4 in a 384-well polypropylene plate. Each sample was tested in quadruplicate. Sample mixtures were incubated at 37°C for 2 hours. During this incubation, streptavidin-coated MSD plates were incubated in MSD blocking buffer for 1 hour. MSD plates were washed, and the sample mixtures were incubated at room temperature for 3 hours. ADAs present in the serum served to bridge the biotinylated and Sulfo-tag–labeled CIS43LS, which was bound to the streptavidin-coated MSD plate. After the incubation, the MSD plate was washed, and MSD read buffer was added. ECL was measured using an MSD Sector S600 plate reader. The test sample was tier 1 positive if the ECL count was greater than the plate-based floating positivity cut point (negative control ECL × 1.196).

Tier 1 positive samples were tested in the tier 2 assay. For the tier 2 confirmatory assay, sample was preincubated with or without 10 μg/mL of unlabeled CIS43LS. Sample activity was compared as the percent signal reduction in the presence of unlabeled CIS43LS. Serum samples from participants were prediluted at 1:2 with assay diluent and either spiked with CIS43LS or not, then incubated for 1 hour at 37°C. Samples were then processed in the same manner as the tier 1 assay. The test sample was tier 2 positive if the percent reduction in signal was greater than the fixed positivity cut point of 19.4%.

### Pf 18S rRNA RT-qPCR

Methodological details for the blood-stage detection from dried blood spots (DBSs) in the CIS43LS trial using Gen3.5DBS *Plasmodium* 18S RT-qPCR have been reported (14). Briefly, the laboratory that performed the RT-qPCR assays was blinded to the group assignments. RNA was extracted from laser-cut DBSs and eluted by the Abbott m2000sp system using the mSample RNA preparation kit (Abbott Molecular). Eluted RNA was combined with SensiFAST Probe Lo-ROX One-Step Kit master mix (Meridian Bioscience), and RT-qPCR was performed on the Abbott m2000rt. The RT-qPCR primers and probes used to target the *Pf* 18S rRNA have been previously described (14), and reactions were performed under thermocycling conditions of 10 minutes at 45°C, 2 minutes at 95°C, followed by 40 cycles of 5 seconds at 95°C and 35 seconds at 54°C. Absolute quantification was determined using an Armored RNA calibrator encoding the full-length *Pf* 18S rRNA.

### Determination of total IgG and IgG1 in serum

Total IgG and IgG1 concentrations were measured using a sandwich-type enzyme-linked immunosorbent assay kit as per the manufacturer’s instructions (Thermo Fisher Scientific). Briefly, 96-well plates were coated either anti-human IgG or anti-human IgG1 capture antibody followed by serum diluted in assay buffer (1:500,000 for IgG and 1:2,500 for IgG1) and then anti-human IgG or anti-human IgG1 antibody conjugated with horseradish peroxidase, with appropriate washes between each step. Wells were incubated with tetramethyl-benzidine substrate solution for 30 minutes (IgG) or 10 minutes (IgG1) before the reaction was stopped with 1 M phosphoric acid (stop solution). Absorbance of each microwell was read on a spectrophotometer at 450 nm. Absolute concentrations were determined from a standard curve of absorbance for 2-fold serially diluted human IgG or human IgG1 samples of known concentrations run simultaneously.

### Pf proteome microarray

The proteome microarray platform technology at Antigen Discovery Inc. was used to fabricate whole proteome antigen arrays for profiling antibodies. The whole proteome antigen array contained 8,871 full-length or fragmented proteins representing 5,233 unique genes from the Pf3D7 genome, or approximately 99% of the Pf3D7 protein-coding genome. In a second experiment, a down-selected proteome microarray containing approximately 1,000 *Pf* proteins (Pf1000 chips) was used to probe posttreatment samples incubated with an anti-idiotypic antibody to prevent residual CIS43LS from binding to the array proteins.

#### Proteome microarray construction.

Proteins were expressed using an in vitro transcription and translation (IVTT) system, the *Escherichia coli* cell-free Rapid Translation System kit (5 Prime). A library of *Pf* partial or complete ORFs cloned into a T7 expression vector pXI was established at Antigen Discovery Inc. This library was created through an in vivo recombination cloning process with PCR-amplified Pf ORFs, and a complementary linearized expressed vector transformed into chemically competent *E*. *coli* was amplified by PCR and cloned into pXI vector using a high-throughput PCR recombination cloning method described elsewhere (59). Each expressed protein includes a 5′ polyhistidine (HIS) epitope and 3′ HA epitope. After expressing the proteins according to the manufacturer’s instructions, translated proteins were printed onto nitrocellulose-coated glass AVID slides (Grace Bio-Labs) using an Omni Grid Accent robotic microarray printer (Digilabs). Microarray chip printing and protein expression were quality checked by probing random slides with anti-HIS and anti-HA mAb with fluorescent labeling.

#### Sample probing.

Serum samples were diluted 1:50 in a 3 mg/mL DH5a *E*. *coli* lysate solution in protein arraying buffer (Maine Manufacturing) and incubated at room temperature for 30 minutes. Chips were rehydrated in blocking buffer for 30 minutes. Blocking buffer was removed, and chips were probed with preincubated serum samples using sealed, fitted slide chambers to ensure no cross-contamination of sample between pads. Chips were incubated overnight at 4°C with agitation. Chips were washed 5 times with TBS–0.05% Tween 20, followed by incubation with mouse anti-human IgG1 Fc (AbCam, catalog AB1927-1001) and rabbit anti-human IgG3 (RevMab Biosciences, catalog 31-1021-00). Chips were again washed and incubated with Cy3 anti-rabbit IgG (Jackson ImmunoResearch Laboratories, catalog 111-165-144) and Cy5 anti-mouse IgG (Jackson ImmunoResearch, catalog 115-175-071). Chips were washed 3 times with TBS–0.05% Tween 20, 3 times with TBS, and once with water. Chips were air-dried by centrifugation at 1,000*g* for 4 minutes and scanned on a ScanArray Express HT spectral scanner (PerkinElmer), and spot and background intensities were measured using an annotated grid file (GAL). Data were exported in Microsoft Excel. The CIS43 mAb was probed at a 0.5 μg/mL concentration and assayed as above using goat DyLight 650–conjugated anti-human IgG (Fortis Life Sciences, Bethyl catalog A80-104D5) for detection of CIS43 binding. In a second experiment, all 160 samples were probed on Pf1000 chips as described above, where the day 168 samples from the CIS43LS group were probed with or without preincubation with 10 μL anti-idiotype blocking antibody.

### Pf proteome microarray data processing and analysis

Raw spot and local background fluorescence intensities, spot annotations, and sample phenotypes were imported and merged. Foreground spot intensities were adjusted by local background by subtraction, and negative values were converted to 1. Next, all foreground values were transformed using the base 2 logarithm (log_2_). The dataset was normalized to remove systematic effects by subtracting the median signal intensity of the IVTT controls for each sample. Since the IVTT control spots carry the chip, sample, and batch-level systematic effects, but also antibody background activity to the IVTT system, this procedure normalizes the data and provides a relative measure of the specific antibody binding to the nonspecific antibody binding to the IVTT controls (i.e., background). With the normalized data, a value of 0.0 means that the intensity is no different than the background, and a value of 1.0 indicates a doubling with respect to background. A seropositivity threshold was established for serum responses as 2 times the IVTT background, or a normalized signal of 1.0. Reactive antigens were defined as those that had seropositive responses in at least 25% of the study population, at either day 0 or day 168 time points. Nonreactive antigens were filtered before group comparisons were performed. For analysis of CIS43 mAb, a conservative reactivity threshold of 2.0 normalized signal intensity, or 4 times the IVTT background, was used to identify non-CSP off-target binding.

### Statistics

Clinical trial data were collected with DFexplore 2023 (v.5.7.0), which secures data with AES 256 encryption; is fully compliant with HIPAA, GDPR, and FDA 21 CFR Part 11 regulations; and is ISO 9001:2015 certified. Data analysis and data visualization were done in R version 4.4.1 and MonolixSuite 2024R1 (see below). Data visualization was performed using the R packages *ggplot2*, *ggpubr*, *cowplot*, *survminer*, and *gtsummary*. Tests used to determine statistical significance of differences are indicated in the figure legends, with the threshold for significance set at *P* < 0.05.

#### PK analysis.

Observed concentration-time data were used to directly calculate C_max_ and time to C_max_. Individual-level noncompartmental analysis was performed using the PKNCA package (60). The AUC was determined using the linear trapezoidal method with linear interpolation by integrating the AUC from infusion to last observed concentration (AUC_0-Clast_) or, when needed, further extrapolated from the last observed concentration (e.g., AUC_0-168d_ or AUC_inf_ _observed_). To estimate population PK parameters, a 2-compartment, proportional error model with administration by 30-minute intravascular infusion was performed on the 220 adult recipients of CIS43LS in the Malian efficacy trial in MonolixSuite 2024R1 and the Monolix R API (61). *t*_1/2β_ was calculated as follows:
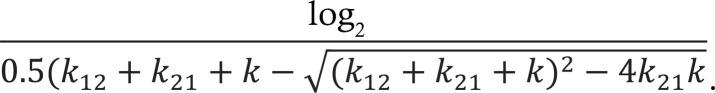


Flow (CL and intercompartmental clearance [Q]) and volume (Vc and peripheral volume [Vp]) parameters were modeled as random effects with interindividual variability (ω) for each estimated by the stochastic approximation expectation-maximization (SAEM) algorithm. Log likelihood was estimated by importance sampling. Correlations between the random effects for CL, Q, Vc, and Vp were included in the model. The individual base models included weight as covariate for each PK parameter,
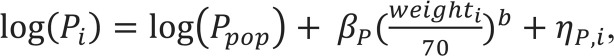


where the allometric exponent *b* was used to normalize to a standard 70 kg weight for flow (*b* = 0.85) and volume (*b* = 1.0) parameters (15, 62). The β coefficients were initially estimated from the base model and then fixed to these values for subsequent models (Table 1). Dose, sex, age, hemoglobin, platelet count, white blood cell count, absolute neutrophil count, and malaria-associated covariates (normalized malaria incidence at time of infusion, *Pf* RT-qPCR positivity at enrollment, serum IgG concentration, and serum IgG1 concentration at enrollment) were formally evaluated as covariates on the CL and Vc parameters using forward selection followed by backward elimination using the following function form:
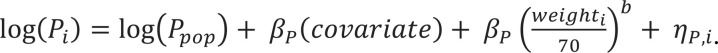


Covariates were retained if inclusion of the single covariate reduced the objective function value by > 3.84 relative to the base model, the relative standard error of parameter was < 60%, the condition number (the ratio of the largest eigenvalue of the correlation matrix over the smallest value) was < 100, and the parameter values converged. A full model with all retained covariates was then evaluated by elimination of each covariate and evaluating the objective function value relative to the base model, corrected Bayesian information criterion relative to both the base model and the model that is more complex by 1 covariate, condition number (< 100), relative standard error of each included covariate, and convergence of parameters. The final model included age (centered by subtracting the minimum age) and serum IgG1 concentration at baseline (centered on the weighted mean) as covariates on the CL parameter and sex and dose as covariates on the Vc parameter. The variance-covariance matrix was fully specified for estimated parameters using the inverse of the observed Fisher information matrix *I*,
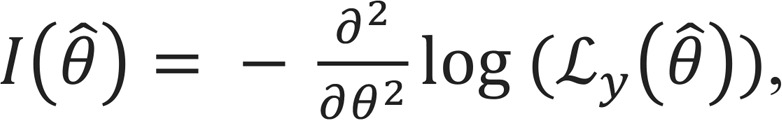


where 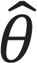 is the population parameter estimate of the unknown parameter *θ*, 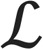 is the log likelihood, and *y* is the observed data. Parameter estimates from SAEM and bootstrap simulations (1,000 replicates) were reported for the final model.

#### Association of protective efficacy with CIS43LS concentration.

To evaluate the association of protective efficacy with CIS43LS concentration, we assessed the risk of *Pf* infection (blood smear positivity) by time-to-first-event analysis in participants enrolled in the Malian efficacy study. For the first analysis, Cox regression was performed to evaluate the association with CIS43LS exposure using the CIS43LS AUC as a time-varying covariate, where CIS43LS AUC was defined as the AUC from CIS43LS administration up to each evaluation time point and 2 time-invariant covariates: *Pf* RT-qPCR positivity at enrollment (prior to drug clearance) as a surrogate for individual malaria risk and baseline IgG1 serum concentration dichotomized using the 66.7 percentile.

For the second analysis, to account for liver-stage development and the prepatent period, we used the CIS43LS concentration 7 days before each *Pf* evaluation, obtained either by interpolation of observed concentrations or using concentrations predicted from the PopPK model. Since the concentration level of CIS43LS was the variable of interest, the 2 CIS43LS dosing arms were combined. Cox regression was performed with the randomization arm (mAb or placebo, time-fixed) and time-varying mAb concentration at 7 days before each *Pf* evaluation (denoted by) as regressors in the following model:
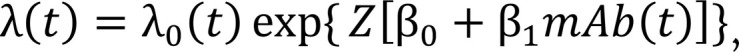


where *Z* denotes treatment indicator (*Z* = 1 for CIS43LS and *Z* = 0 for placebo). The model assumes that, given the CIS43LS concentration level, the hazard ratio of infection under CIS43LS versus placebo is constant. The protective efficacy corresponding to a specific mAb concentration is thus,
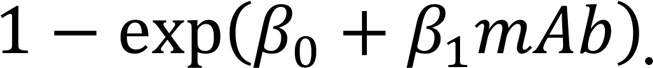


The proportional hazards assumptions were evaluated on the scaled Schoenfeld residuals.

### Study approval

The CIS43LS trial was conducted in accordance with International Council for Harmonisation Good Clinical Practice guidelines and applicable regulations in Mali. The US FDA reviewed the trial protocol in the investigational new drug application (IND 147485), sponsored by the National Institute of Allergy and Infectious Diseases (NIAID). The protocol and informed consent forms were approved by the ethics committee at Faculté de Médecine et d’Odonto-Stomatologie and Faculté de Pharmacie at the University of Sciences, Techniques and Technologies of Bamako and by Malian national regulatory authorities. Community permission was obtained from participating sites. All participants provided written informed consent.

### Data availability

Underlying data are available in the Supporting Data Values file and from the corresponding author upon request. Supporting analytic code for the manuscript can be accessed at https://github.com/TranLab/CIS43LS-PKPD-Malian-Adults (commit ID 88339ff).

## Author contributions

ZH, KK, A Ongoiba, SAH, SD, DD, ACP, BT, RAS, and PDC designed the clinical trial. KK, A Ongoiba, SAH, SD, ACP, and PDC supervised the clinical trial. A Ongoiba, HC, JD, SD, DD, ACP, SL, MEP, ST, EEC, and AD coordinated the operational conduct of the clinical trial and gathered and managed the clinical trial data and biospecimens. SCM generated the RT-qPCR data. HC and BHC generated the IgG and IgG1 data. RS, KL, and RK generated the PK and ADA data, which LS, SRN, and BL supervised. A Oberai, ADS, and JJC generated the protein microarray data. TMT, ZH, SJ, NA, JS, EVC, and PDC analyzed the data. TMT and PDC wrote the manuscript. ZH, KK, A Ongoiba, SJ, NA, SAH, JS, LS, EVC, BT, and RAS reviewed and edited the manuscript. All authors reviewed the manuscript.

## Conflict of interest

RAS has a filed patent (PCT/US2018/017826) describing the CIS43 antibody.

## Funding support

This research was supported in part by the Intramural Research Program of the NIH and is subject to the NIH Public Access Policy. Through acceptance of this federal funding, the NIH has been given a right to make the work publicly available in PubMed Central.

- Vaccine Research Center, NIAID, NIH.
- NIH Intramural Research Program.
- NIAID grant 1R01AI192628-01 (to TMT).
- Gates Foundation (INV-088153 to TMT).

## Supplementary Material

Supplemental data

ICMJE disclosure forms

Supplemental data set 1

Supporting data values

## Figures and Tables

**Table 1:**
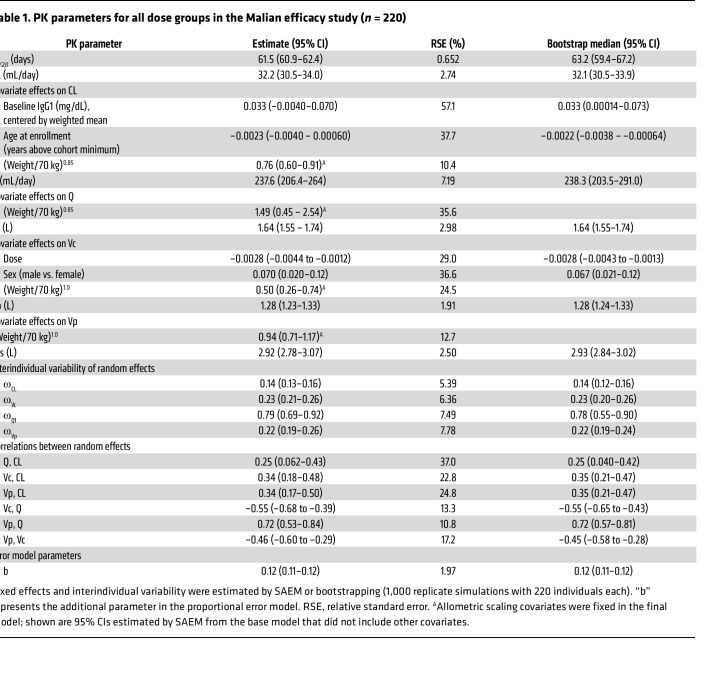
PK parameters for all dose groups in the Malian efficacy study (*n* = 220)

## Version 1 07/06/2026

In-Press Preview

## Version 2 08/17/2026

Electronic publication
