# Supplementary material for: Pharmacokinetics and pharmacodynamics of a long-acting monoclonal antibody against malaria in African adults
Source: J Clin Invest. 2026 Jul 2;136(16):e207559. doi: 10.1172/JCI207559 (PMC13476135; doi:10.1172/JCI207559)
Supplement: Supplementary file 1 [file jci-136-207559-s109.pdf]

Supplementary material

Title:
Pharmacokinetics and pharmacodynamics of a long-acting monoclonal antibody against malaria in African adults

Authors:
Tuan M. Tran[1,2], Zonghui Hu[3], Kassoum Kayentao[4], Aissata Ongoiba[4], Sam Jones[5], Nada Abla[5], Sara A. Healy[6], Hamidou Cisse[6], Bickey H. Chang[6], Jeff Skinner[6], Leonid Serebryannyy[7], Sandeep Narpala[7], Robin Carroll[7], Kwang Low[7], Rachel Kazmierski[7], Bob C. Lin[7], Joana Dias[7], Safiatou Doumbo[4], Didier Doumtabe[4], Anne C. Preston[6], Shanping Li[6], Mary E. Peterson[6], Amit Oberai[8], Adam D. Shandling[8], Joseph J. Campo[8], Sean C. Murphy[9,10], Shinyi Telscher[7], Emily E. Coates[7], Edmund V. Capparelli[11], Amagana Dolo[4], Boubacar Traore[4], Robert A. Seder[7], Peter D. Crompton[6]

Affiliations:
[1]Division of Infectious Diseases, Department of Medicine, Indiana University School of Medicine, Indianapolis, IN, USA.
[2]Ryan White Center for Pediatric Infectious Disease and Global Health, Department of Pediatrics, Indiana University School of Medicine, Indianapolis, IN, USA.
[3]Office of Biostatistics Research, National Institute of Allergy and Infectious Diseases, National Institutes of Health, Bethesda, MD, USA.
[4]Malaria Research and Training Centre, Mali International Center of Excellence in Research, University of Sciences, Techniques and Technologies of Bamako, Bamako, Mali.
[5]MMV Medicines for Malaria Venture, Geneva, Switzerland.
[6]Malaria Infection Biology and Immunity Section, Division of Intramural Research, National Institute of Allergy and Infectious Diseases, National Institutes of Health, Rockville, MD, USA.
[7]Vaccine Research Center, National Institute of Allergy and Infectious Diseases, National Institutes of Health, Bethesda, MD, USA.
[8]Antigen Discovery, Incorporated, Irvine, CA, USA.
[9]Department of Laboratory Medicine and Pathology and Center for Emerging and Re-emerging Infectious Diseases, University of Washington, Seattle, WA, USA.
[10]Department of Laboratories, Seattle Children's Hospital, Seattle, WA, USA.
[11]Departments of Pediatrics and Pharmacy, UC San Diego Schools of Medicine and Skaggs School of Pharmacy and Pharmaceutical Sciences, La Jolla, CA, USA.

	C_{max} (µg/mL)	T_{max} (hours)	C_{168d} (µg/mL)*	C_{252d} (µg/mL)**	$AUC_{0\text{-}168d}$ (µg/mL)days*
Dose Group	Mean (SD)				
5 mg/kg IV (n=6)	121.0 (22.41)	0.56 (0.040)	8.01 (1.09)	3.03 (0.594)	4365 (933.2)
10 mg/kg IV (n=116)	241.7 (62.36)	1.9 (5.3)	16.6 (3.98)	7.13 (1.50)	8326 (1146)
40 mg/kg IV (n=116)	1032 (286.2)	0.60 (0.11)	65.4 (14.8)	27.1 (6.80)	33530 (4872)

Supplemental Table 1. PK parameters by dose group. C_{max} = maximum serum concentration; T_{max} = time to maximum serum concentration; C_{168d}, C_{252d} = serum concentration on day 168 and 252, respectively. $AUC_{0\text{-}168d}$ = area under the curve from day 0 to day 168.

*Analysis excludes 14 participants in the efficacy study (Part B) who did not have samples up to day 168.

** Analysis only includes participants in the dose escalation study (Part A) who received CIS43LS 5 mg/kg IV (n=6), 10 mg/kg IV (n=6), or 40 mg/kg IV (n=6).

dose	variable	strata	protected, median (IQR)	infected, median (IQR)	estimate	p value
10 mg/kg	Pf status at enrollment	Pf (+) at enrollment	9500 (9070 - 10300)	8440 (7910 - 9690)	-977.38	0.034*
10 mg/kg	Pf status at enrollment	Pf (-) at enrollment	9710 (9390 - 10700)	10100 (8850 - 11400)	244.33	0.71
10 mg/kg	IgG1 baseline concentration	bottom 33%	9820 (9230 - 11000)	8960 (8470 - 11400)	-486.88	0.38
10 mg/kg	IgG1 baseline concentration	middle 33%	9640 (9340 - 10400)	10400 (9570 - 11500)	632.10	0.24
10 mg/kg	IgG1 baseline concentration	top 33%	9590 (9010 - 10100)	8300 (7760 - 8990)	-1096.27	0.027*
40 mg/kg	Pf status at enrollment	Pf (+) at enrollment	39500 (35300 - 42500)	38500 (33000 - 39900)	-2598.33	0.22
40 mg/kg	Pf status at enrollment	Pf (-) at enrollment	38900 (35900 - 45200)	37400 (34600 - 44800)	-1745.26	0.53
40 mg/kg	IgG1 baseline concentration	bottom 33%	40100 (38000 - 43700)	38600 (37500 - 40500)	-1538.08	0.40
40 mg/kg	IgG1 baseline concentration	middle 33%	40300 (36700 - 44900)	42500 (37500 - 43400)	-219.52	0.88
40 mg/kg	IgG1 baseline concentration	top 33%	36700 (34300 - 41400)	35600 (30200 - 37500)	-3106.67	0.28

Supplemental Table 2. Comparison of CIS43LS AUC values between protected and infected participants stratified by *Pf* infection status at enrollment and total serum IgG1 concentration at baseline. Related to Figure 2. Median $AUC_{inf\ observed}$ (from 0 to infinity, extrapolated from the last observed concentrations) values and interquartile ranges (IQR) are shown. Statistical significance was determined by Wilcoxon signed-rank test.

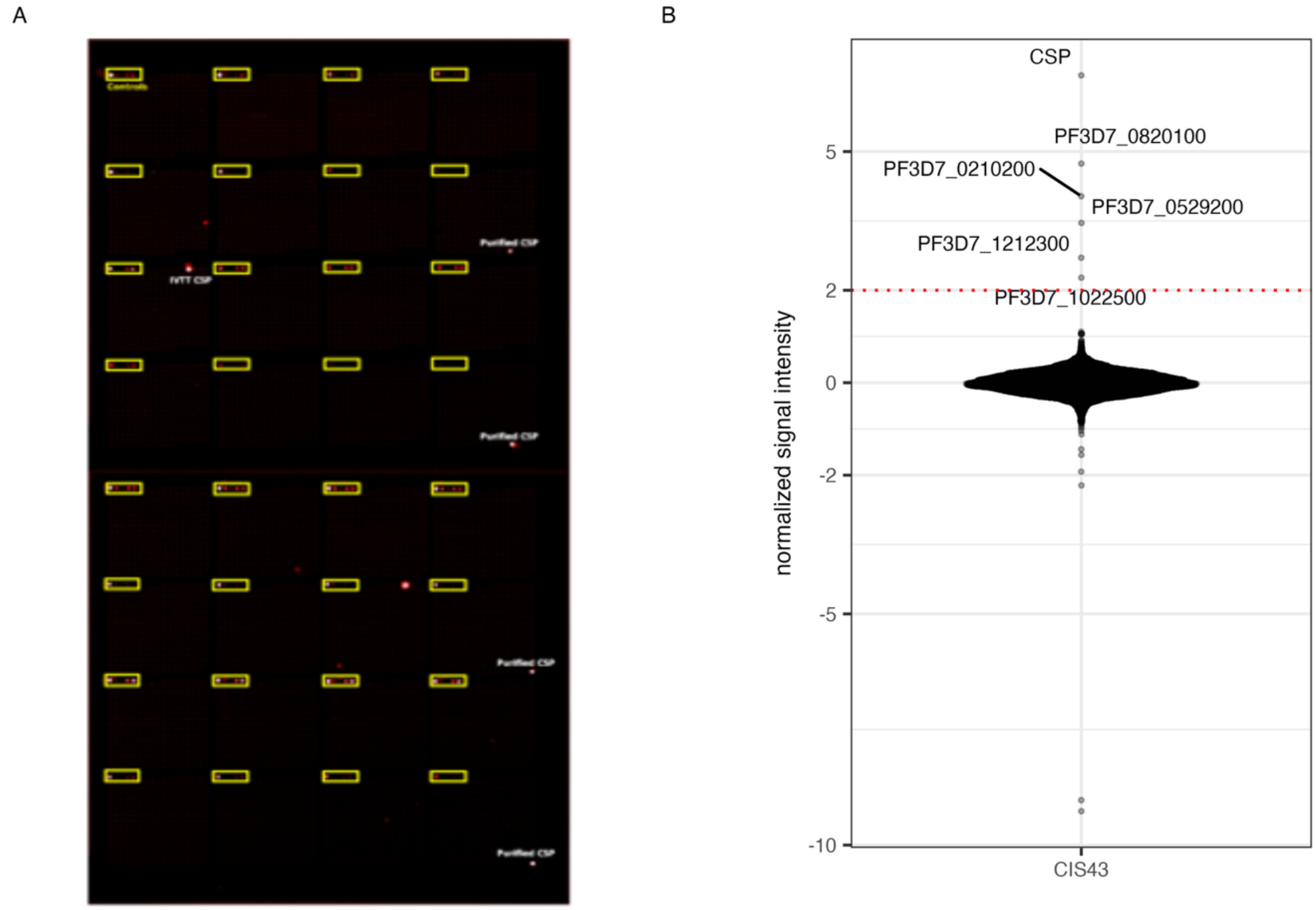

C

Gene ID	Description	Signal Peptide	Transmembrane Domain
PF3D7_0820100	RNA-binding protein, putative	yes	no
PF3D7_0210200	conserved Plasmodium protein, unknown function	no	no
PF3D7_0529200	sugar transporter, putative	no	yes
PF3D7_1212300	WD repeat-containing protein, putative	no	no
PF3D7_1022500	citrate synthase, mitochondrial, putative	no	no

Supplemental Figure 1. CIS43 binding to non-CSP *Pf* proteins. **(A)** The microarray slide scan shows CIS43 probed on a *Pf* whole proteome microarray. Purified recombinant CSP and cell-free in vitro expressed (IVTT) CSP spots are labeled in white text, and array controls are marked in yellow borders. Approximately 5 non-CSP IVTT proteins were visibly reactive with CIS43. **(B)** Beeswarm plot shows the distribution of normalized signal intensity on the array for CIS43. The red line represents a conservative reactivity cut-off of 2.0 normalized signal intensity, or approximately 4-fold the array spot background. Proteins above the reactivity threshold and the CSP are labeled with PF3D7 gene IDs. **(C)** Description of *Pf* proteins labeled in B.

group 1	group 2	P value
CIS43LS AUC, lower 33%	CIS43LS AUC, middle 33%	0.017
CIS43LS AUC, lower 33%	CIS43LS AUC, upper 33%	0.0040
CIS43LS AUC, middle 33%	CIS43LS AUC, upper 33%	0.59

Supplemental Table 3. Significance testing to compare CIS43LS AUC tercile survival curves in Figure 4A. Non-parametric two-way comparisons between CIS43LS AUC terciles were performed using the Peto & Peto modification of the Gehan-Wilcoxon test to determine whether differences in survival curves in Figure 4A were significant.

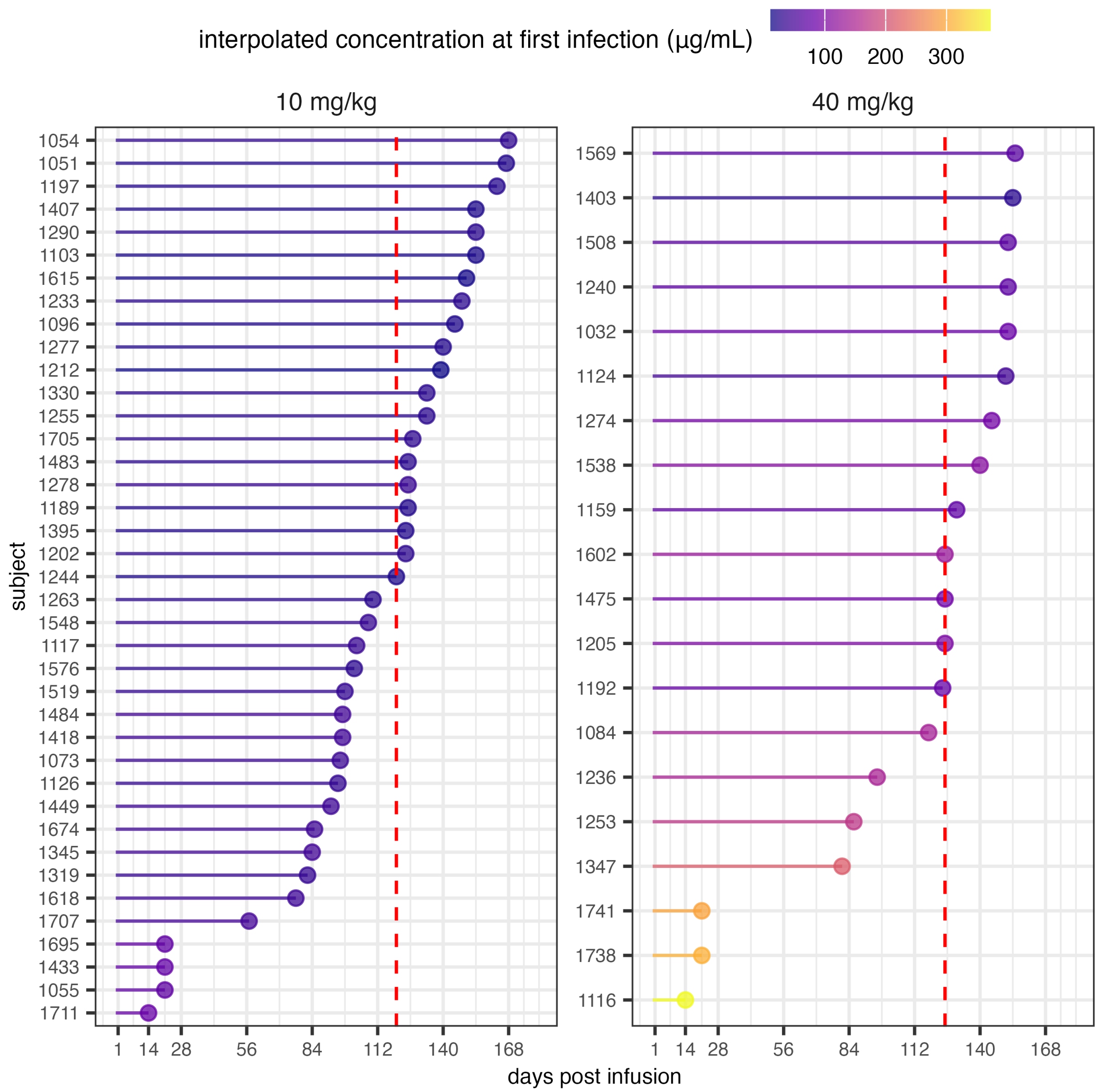

Supplemental Figure 2. Relationship between CIS43LS concentration with time-to-first *Pf* infection by participant. Plots showing relationship between time of first positive blood smear and interpolated CIS43LS concentration 7 days before infection for participants in the 10 mg/kg (left) and 40 mg/kg (right) groups. Red dashed lines represent the median days to positive blood smear.

Variable	Dose<br>**10 mg/kg** N = 110[1]	 **40 mg/kg** N = 110[1]
Site		
Kalifabougou	67 / 110 (61%)	67 / 110 (61%)
Torodo	43 / 110 (39%)	43 / 110 (39%)
Sex		
female	51 / 110 (46%)	47 / 110 (43%)
male	59 / 110 (54%)	63 / 110 (57%)
Age (years)		
Median (Q1, Q3)	34 (27, 41)	35 (27, 43)
Min, Max	18, 54	18, 53
Weight (kg) at infusion		
Median (Q1, Q3)	64 (58, 70)	62 (55, 67)
Min, Max	44, 94	43, 101
CrCl (mL/min)		
Median (Q1, Q3)	81 (72, 94)	80 (73, 91)
Min, Max	56, 114	55, 122
Absolute neutrophil count (X1000 cells/µl)		
Median (Q1, Q3)	2.58 (1.96, 3.40)	2.54 (1.97, 3.08)
Min, Max	0.64, 6.09	1.02, 5.62
White blood cell count (X1000 cells/µl)		
Median (Q1, Q3)	5.60 (4.60, 6.40)	5.50 (4.70, 6.10)
Min, Max	2.20, 9.90	2.80, 10.80
Hemoglobin (g/dL)		
Median (Q1, Q3)	13.40 (12.30, 14.50)	13.40 (12.50, 14.20)
Min, Max	9.90, 17.30	8.40, 16.20
Platelet count (X1000 cells/µl)		
Median (Q1, Q3)	235 (209, 276)	242 (199, 288)
Min, Max	118, 404	105, 441
log(normalized malaria incidence at infusion)		
Median (Q1, Q3)	13 (3, 37)	13 (3, 35)
Min, Max	1, 55	1, 55
Pf qRT-PCR status at enrollment		
negative	65 / 107 (61%)	63 / 108 (58%)
positive	42 / 107 (39%)	45 / 108 (42%)
not determined	3	2
Baseline serum IgG concentration (mg/dL)		
Median (Q1, Q3)	1,310 (1,081, 1,632)	1,218 (947, 1,480)
Min, Max	544, 3,486	683, 2,569
Baseline serum IgG1 concentration (mg/dL)		
Median (Q1, Q3)	1,137 (870, 1,454)	1,136 (906, 1,602)
Min, Max	438, 2,685	574, 2,816

[1]n / N (%)

Supplemental Table 4. Demographic, anthropometric, hematologic, and malariometric indices evaluated as potential covariates in the PopPK model.

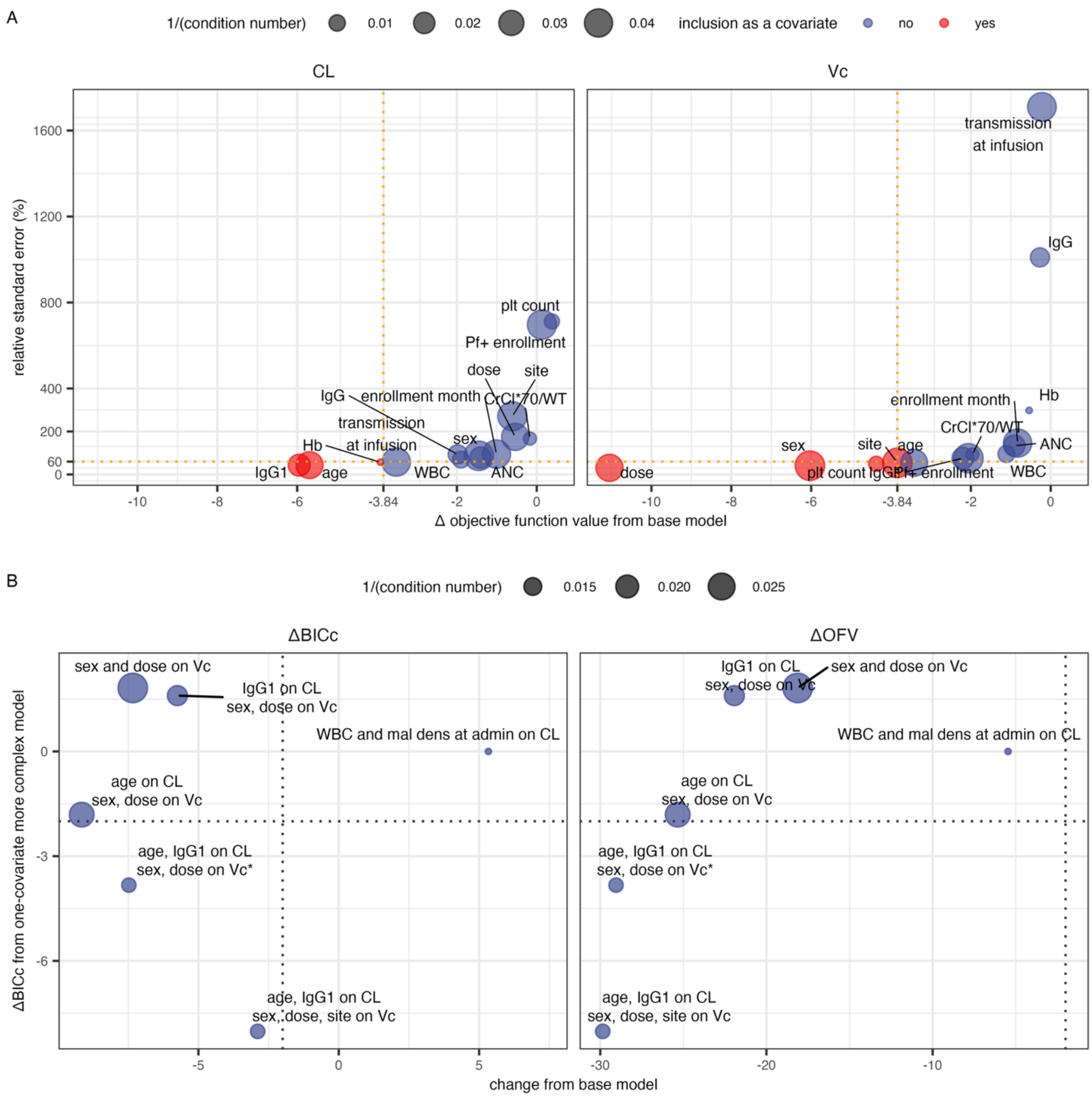

Supplemental Figure 3. Evaluation of covariates on PK parameters by forward selection and backward elimination. (A) Plots showing the relative standard error (RSE) for the indicated covariate when added as a single covariate to either the clearance (CL) or central volume (Vc) parameter in a base population PK model that included allometric scaling on CL, Q1, Vc and Vp versus the change (Δ) in objective function value (OFV) relative to this base model. Point size indicates the reciprocal of the condition number. Red covariates were included in the full model. **(B)** Plots showing the change in corrected Bayesian information criteria (BICc) from a model that is more complex by one more covariate versus the change in either BICc or OFV from the base model. Models with condition number >100 are likely to be over-parameterized and are excluded. *Indicates final model chosen based on minimizing OFV, BICc, and condition number while considering relative standard errors and convergence of parameter estimates. Vertical and horizontal dotted lines indicate thresholds for inclusion and exclusion of covariates and models.

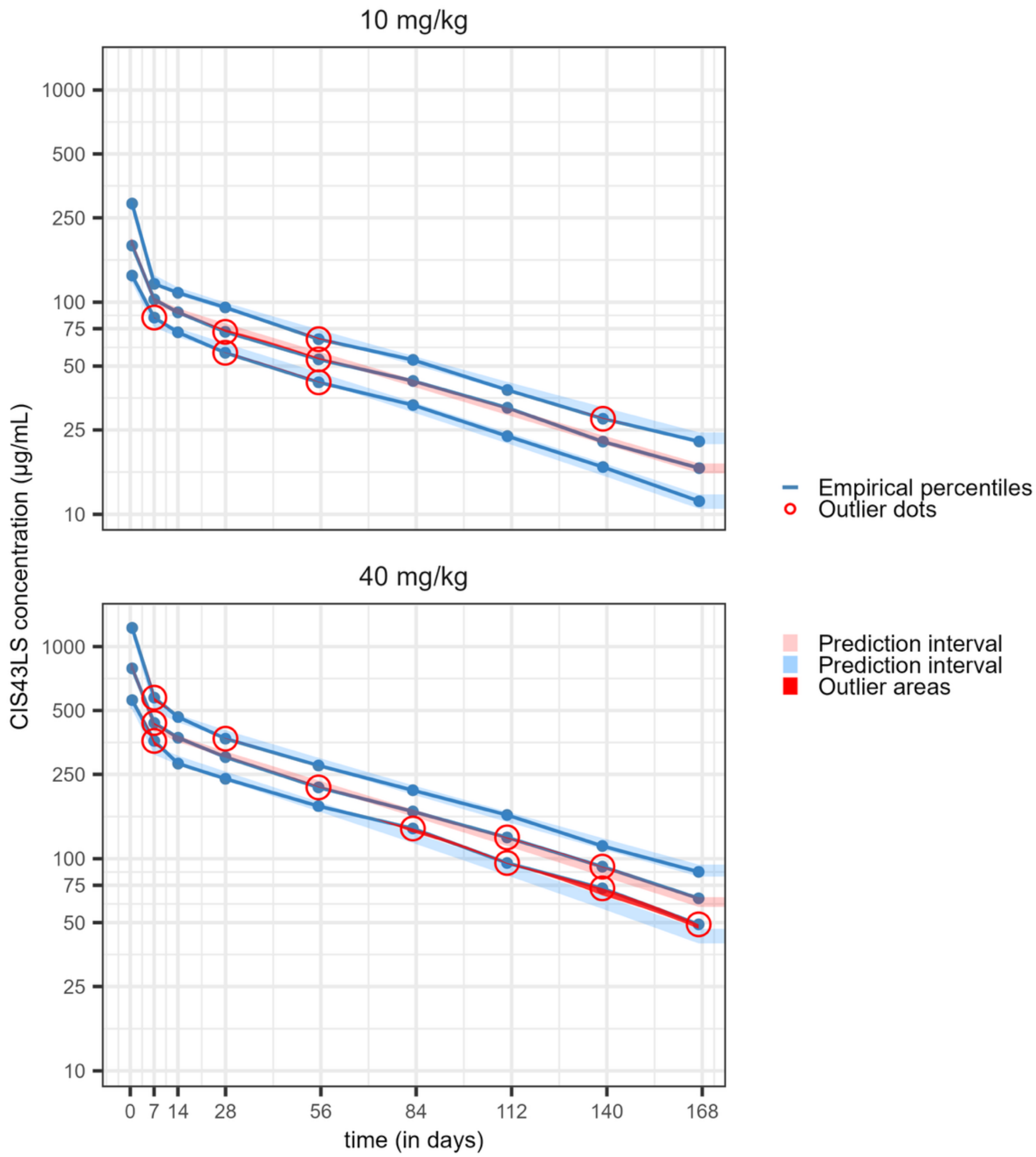

Supplemental Figure 4. Visual predictive check of simulated and empirical percentiles. Shown are 10th-, 50th-, and 90th-percentile prediction intervals of simulated data computed from multiple Monte Carlo simulations of the final PopPK model (3000 simulations with >1000 observations per dose group) along with 10th-, 50th-, and 90th-percentiles of the empirical (observed) data. Outliers are highlighted as red dots and red areas.

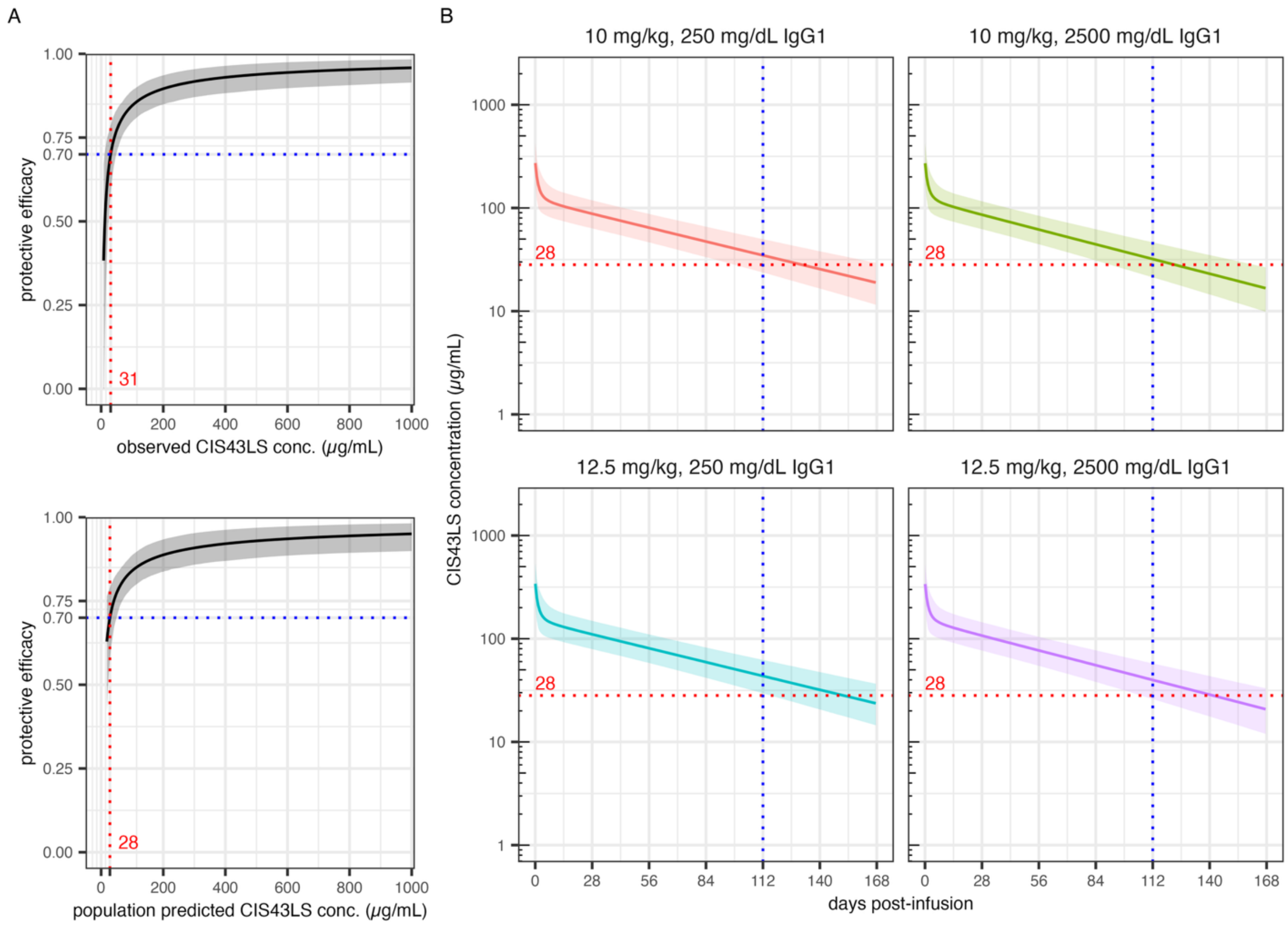

Supplemental Figure 5. CIS43LS doses predicted to provide 70% protective efficacy against *Pf* infection for 3-4 months after administration. (A) Concentration vs. efficacy plot showing the minimum CIS43LS serum concentration required to protect against natural *Pf* infections in the context of intense seasonal transmission at 70% efficacy. Analysis was performed by Cox regression with the randomization arm (CIS43LS or placebo, time-fixed) and estimated CIS43LS concentration (time-varying) as regressors, combining both 10 and 40 mg/kg dose arms. Top and bottom plots used CIS43LS concentrations interpolated from observed values or predicted from the PopPK model, respectively. **(B)** PK simulations of cohorts (1000 individuals/cohort) with baseline serum concentrations of 250 or 2500 mg/dL dosed at either 10 or 12.5 mg/kg CIS43LS using the PopPK model derived from the Malian efficacy trial. For each simulated cohort, the curve represents the median concentration with 2.5%-ile and 97.5%-ile bands of 100 replicates. Red and blue dotted lines represent required CIS43LS concentration required for 70% efficacy determined in panel A and the WHO-preferred duration for anti-malaria mAb efficacy of ~4 months, respectively.

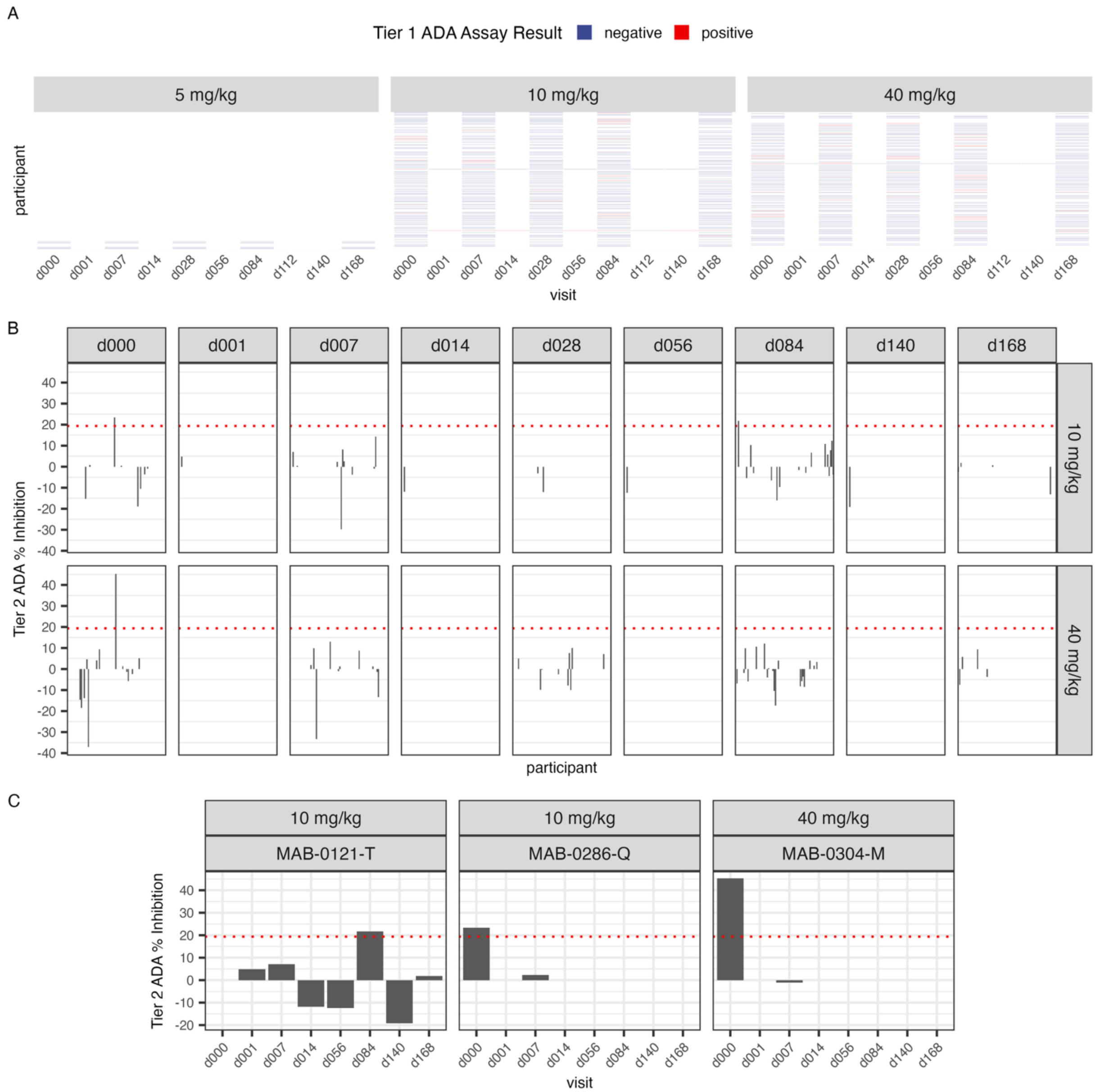

Supplemental Figure 6. Anti-drug antibody data for **(A)** Tier 1 and **(B)** Tier 2 assays. **(C)** Longitudinal Tier 2 data for participants with at least one Tier 2 assay result that was above the positivity cut-off indicated by the dotted red line (19.36 %). Only Tier 1 positive samples were tested in the Tier 2 assay.
